# YTHDF2 promotes mitotic entry and is regulated by cell cycle mediators

**DOI:** 10.1371/journal.pbio.3000664

**Published:** 2020-04-08

**Authors:** Qili Fei, Zhongyu Zou, Ian A. Roundtree, Hui-Lung Sun, Chuan He

**Affiliations:** 1 Department of Chemistry, The University of Chicago, Chicago, Illinois, United States of America; 2 Howard Hughes Medical Institute, The University of Chicago, Chicago, Illinois, United States of America; 3 Guangdong Laboratory for Lingnan Modern Agriculture, Genome Analysis Laboratory of the Ministry of Agriculture, Agricultural Genomics Institute at Shenzhen, Chinese Academy of Agricultural Sciences, Shenzhen, Guangdong, China; 4 Medical Scientist Training Program, The University of Chicago, Chicago, Illinois, United States of America; 5 Department of Biochemistry and Molecular Biology, The University of Chicago, Chicago, Illinois, United States of America; 6 Institute for Biophysical Dynamics, The University of Chicago, Chicago, Illinois, United States of America; Yale University, UNITED STATES

## Abstract

The *N*^6^-methyladenosine (m^6^A) modification regulates mRNA stability and translation. Here, we show that transcriptomic m^6^A modification can be dynamic and the m^6^A reader protein YTH *N*^6^-methyladenosine RNA binding protein 2 (YTHDF2) promotes mRNA decay during cell cycle. Depletion of YTHDF2 in HeLa cells leads to the delay of mitotic entry due to overaccumulation of negative regulators of cell cycle such as Wee1-like protein kinase (WEE1). We demonstrate that WEE1 transcripts contain m^6^A modification, which promotes their decay through YTHDF2. Moreover, we found that YTHDF2 protein stability is dependent on cyclin-dependent kinase 1 (CDK1) activity. Thus, CDK1, YTHDF2, and WEE1 form a feedforward regulatory loop to promote mitotic entry. We further identified Cullin 1 (CUL1), Cullin 4A (CUL4A), damaged DNA-binding protein 1 (DDB1), and S-phase kinase-associated protein 2 (SKP2) as components of E3 ubiquitin ligase complexes that mediate YTHDF2 proteolysis. Our study provides insights into how cell cycle mediators modulate transcriptomic m^6^A modification, which in turn regulates the cell cycle.

## Introduction

Methyltransferase Like 3 (METTL3) and METTL14 [1]—together with several key components, such as Wilms tumor 1-associated protein (WTAP) [2], Vir Like m^6^A Methyltransferase Associated (VIRMA) [3], and Zinc Finger CCCH-Type Containing 13 (ZC3H13) [4]—form a methyltransferase complex to mediate *N*^6^-methyladenosine (m^6^A) modification on mRNAs in a co-transcriptional manner [5,6]. m^6^A, the most abundant modification on mRNAs, plays multifaceted roles in regulating pre-mRNA processing [7,8], nuclear export [9], stability [10], translation [11], and other biochemical properties [12,13] of mRNA in eukaryotes. The myriad roles of the m^6^A modification mostly rely on downstream RNA-binding proteins, known as m^6^A “readers,” that preferentially recognize m^6^A-modified RNAs [9–11,14,15].

YTH *N*^6^-methyladenosine RNA binding protein 2 (YTHDF2) was the first functionally verified m^6^A reader to promote the degradation of m^6^A-modified mRNAs in humans [10]. It accelerates the decay of m^6^A-marked transcripts by directly recruiting the Carbon Catabolite Repressor 4-Negative on TATA (CCR4-NOT) deadenylase complex [16] or the endoribonucleolytic RNase P/Mitochondrial RNA Processing (MRP) complex [17]. In zebrafish, Ythdf2 promotes the clearance of the maternal transcripts during maternal to zygotic transition (MZT) [18]. Loss of Ythdf2 in zebrafish delays MZT initiation and impedes zygotic genome activation [18]. Likewise, Ythdf2 was confirmed maternally essential for early zygotic development in mice [19], and Ythdf2 depletion also negatively impacts the proliferation and differentiation of neural progenitor cells in mouse [20], suggesting broad impact of Ythdf2 on mRNA degradation in different tissues and cell types in vertebrates. The significant role of YTHDF2 in cell differentiation was further shown with depletion of YTHDF2-blocking differentiation and driving self-renewal of both mouse and human hematopoietic stem cells [21,22]. This protein is also critical to acute myeloid leukemia (AML) [23].

The m^6^A pathway has been implicated in regulating genes related to cell cycle. It was reported that transcripts involved in cell cycle regulation contain m^6^A modification in mouse embryonic stem cells (mESC) [24]. Down-regulation of METTL3 in AML causes cell cycle arrest because of down-regulation of genes in the cell cycle pathway [25]. AlkB Homolog 5 (ALKBH5), an m^6^A demethylase, facilitates glioblastoma proliferation by demethylating the transcripts of forkhead box protein M1 (FOXM1), an important cell cycle regulator [26]. METTL14 was shown to be essential for mammalian cortical neurogenesis by facilitating the decay of a few categories of transcripts, including those regulating the cell cycle [27]. These studies suggest that the m^6^A pathway potentially plays a role in cell cycle regulation; however, mechanistic studies are still limited, and the impact of m^6^A readers on cell cycle remains unexplored. Here, we investigated changes in transcriptomic m^6^A during cell cycle and demonstrated a role of YTHDF2 in regulating phase-specific transcript degradation. Furthermore, we identified the proteolysis pathway of YTHDF2, which establishes a mechanistic interconnection between the m^6^A pathway and cell cycle.

## Results

### Dynamic changes of transcriptomic m^6^A during cell cycle

**Fig 1:**
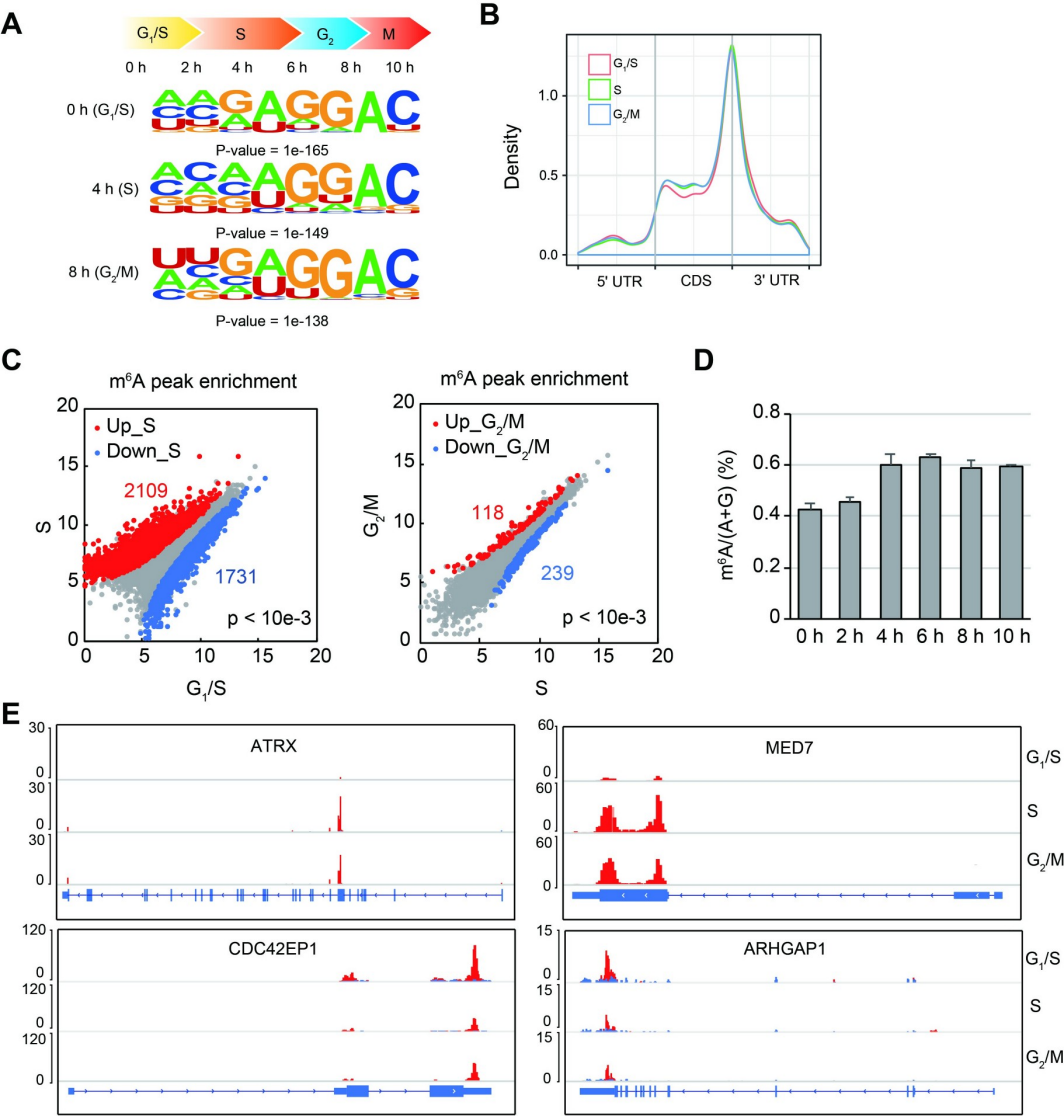
Dynamic transcriptomic m^6^A changes during cell cycle. (A) Enriched RNA motifs from the m^6^A MeRIP-seq data from cells at different phases of cell cycle. (B) Metagene analysis of m^6^A peak distribution on mRNAs. (C) Differential m^6^A enrichment analysis across G_1_/S, S, and G_2_/M phases of cell cycle. (D) The m^6^A levels of mRNA quantified by LC-MS/MS at different time points post G_1_/S synchronization. y-Axis indicates the ratio of m^6^A to the sum of A and G. (E) Examples of genes with dynamic m^6^A modification on mRNAs. *ATRX* and *MED7* m^6^A peak enrichment increases at S phase compared with G_1_/S and are related to DNA duplex unwinding and Pol II transcription, respectively. m^6^A peak enrichment of *CDC42EP1* and *ARHGAP1* reduces at S phase, which are related to Rho protein signaling or GTPase activity. Blue and red bars indicate the input and IP read coverages, respectively. Underlying data for this figure can be found in S1 Data. ARHGAP1, Rho GTPase Activating Protein 1; ATRX, alpha thalassemia/mental retardation syndrome X-linked; CDC42EP1, CDC42 Effector Protein 1; CDS, coding sequence; IP, immunoprecipitation; LC-MS/MS, liquid chromatography–tandem mass spectrometry; MED7, Mediator Complex Subunit 7; m^6^A, *N*^6^-methyladenosine; MeRIP-seq, methylated RNA immunoprecipitation sequencing; Pol II, RNA polymerase II; UTR, untranslated region.

Cells go through dynamic transcriptome changes during cell cycle, especially during G_1_ and S phases [28]. We hypothesized that m^6^A modification may also change during cell cycle because m^6^A on mRNAs are deposited co-transcriptionally [5,6] and are known to affect cell cycle. To understand how transcriptomic m^6^A changes during cell cycle, we synchronized HeLa cells to the stage of G_1_/S transition by double thymidine block and collected cells for m^6^A methylated RNA immunoprecipitation sequencing (MeRIP-seq) from 0, 4, and 8 hours post release, which correspond to G_1_/S, S, and G_2_/M phases, respectively (S1A Fig) (S1 Table). Motif search of m^6^A peaks all identified motifs containing the “GGAC” core motif (Fig 1A). m^6^A peaks predominantly reside on the 3′ untranslated region (UTR) of transcripts at all phases (Fig 1B), consistent with previous reports [29,30]. Gene ontology (GO) enrichment analysis of the m^6^A-marked common genes at 3 phases identified a number of GO terms, including “cell cycle” (S1B Fig), suggesting that the m^6^A pathway regulates genes involved in cell cycle control, consistent with a previous study in mESC [24].

Differential enrichment analysis of m^6^A peaks was performed to assess the changes in m^6^A levels with progression across the 3 cell phases. We found that 2,109 peaks from 1,489 genes showed significantly higher enrichment from G_1_/S to late S phase, while 1,731 peaks from 1,416 genes exhibited reduced enrichment (Fig 1C). In contrast, only 118 and 239 peaks, corresponding to 112 and 210 genes, displayed differential changes from late S to G_2_/M phase (Fig 1C) (S2 Table). Correlation analysis of these samples showed a higher correlation between late S and G_2_/M (S1C Fig). These results suggest that transcriptomic m^6^A is dynamic through G_1_ and S phase while remaining relatively steady from late S to G_2_/M phase.

We collected cells at more time points after synchronization and measured m^6^A levels of mRNA by mass spectrometry. We found that m^6^A level increased from G1/S (0 hours) to S (4 hours) phase and remained steady thereafter (Fig 1D). This observation is consistent with the transcriptomic m^6^A changes during cell cycle (Fig 1C). To test whether the observed m^6^A changes were caused by potential changes of methyltransferase or demethylases, we measured the protein levels of METTL3 and METTL14, members of the core m^6^A methyltransferase complex, and fat mass and obesity-associated protein (FTO), an m^6^A demethylase by western blot. We found that METTL3 showed lower protein level at early G_1_/S (0 h) phase and M (10 h) phase, while METTL14 only decreased at M phase (S1D Fig). FTO remained relatively steady during cell cycle (S1D Fig), indicating that the methyltransferase activity—instead of the demethylase activity—might affect m^6^A levels during cell cycle. In contrast, RNA sequencing (RNA-seq) data (see subsequently) revealed that the transcript levels of METTL3, METTL14, and FTO all remained relatively steady (S1E Fig), suggesting possible translational or post-translational regulation for METTL3 and METTL14 during cell cycle.

Correlation analysis of gene expression level and m^6^A enrichment revealed that the overall gene expression showed a weak negative correlation with m^6^A peak enrichment (S2A Fig), consistent with previous studies [31,32]. To investigate whether gene expression affects m^6^A modification, we next specifically analyzed genes with differential m^6^A enrichment during cell cycle phase transitions. We found that genes with higher m^6^A enrichment in the next phase tend to have significantly higher expression levels; in contrast, genes with reduced m^6^A modifications concurrently showed lower expression (S2B Fig). These results reveal tight correlation between transcriptional regulation and m^6^A modifications during phase transitions, when dynamic transcriptomic changes occur during cell cycle.

To understand the biological pathways undergoing differential m^6^A changes, we performed GO enrichment analysis for the genes exhibiting m^6^A dynamics. Genes such as *ATRX* and *MED7* with increased m^6^A from G_1_/S to late S phase (Fig 1E) were mostly classified as GO terms related to “DNA duplex unwinding” and “regulation of transcription” (S2C Fig), which are typical S phase events [33]. In contrast, terms such as “Rho protein signal” and “GTPase activity” (S2D Fig), which are essential for G_1_ phase [34], showed enrichment for genes with decreased m^6^A enrichment from G_1_/S to late S phase, such as *CDC42EP1* and *ARHGAP1* (Fig 1E). At the G_2_/M phase, “microtubule-based process” showed higher m^6^A enrichment, which is a hallmark of mitosis (S2E Fig) [35]. In addition, genes related to transcription and response to DNA damage, which are active at S phase [33], exhibited a reduction of m^6^A on transcripts (S2F Fig). Collectively, m^6^A profiling at different phases revealed that transcriptomic m^6^A is actively regulated during cell cycle, indicating that m^6^A modification of the phase-specific transcripts might be important for cell cycle progression in HeLa cells that we examined.

### YTHDF2 depletion elevates phase-specific transcripts

**Fig 2:**
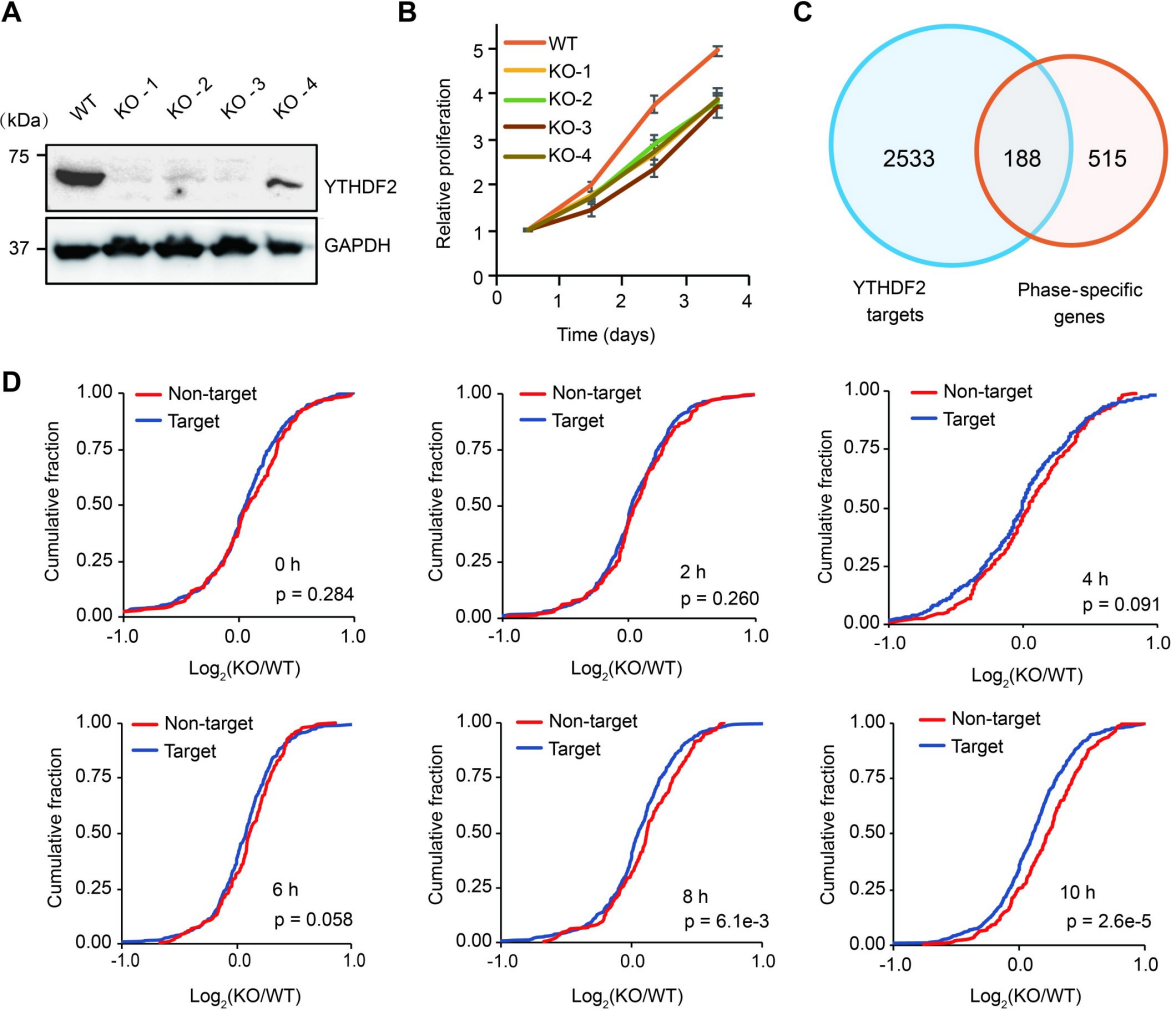
YTHDF2 depletion delays cell proliferation and elevates target transcripts. (A) Western blot of YTHDF2 in CRISPR-Cas9 KO cell lines. (B) Cell proliferation assays of WT and *YTHDF2* KO cell lines. (C) Intersection of genes that are confident YTHDF2 targets and the ones that are phase specific. (D) Cumulative distribution of phase-specific transcripts that are YTHDF2 bound (188 genes, in red) and unbound (515 genes, in blue) by comparing WT and KO cell lines. The x-axes indicate the log2 fold change of gene expression in KO versus WT. *P* values were calculated using the Mann-Whitney test. Underlying data for this figure can be found in S1 Raw Images and S1 Data. GAPDH, glyceraldehyde 3-phosphate dehydrogenase; KO, knockout; WT, wild type; YTHDF2, YTH *N*^6^-methyladenosine RNA binding protein 2.

During cell cycle, the expression of hundreds of genes dynamically fluctuates along with phase transitions in both human and yeast [36–38]. Like transcription, mRNA decay is also dynamic during cell cycle [39]. However, the mechanism underlying mRNA decay remains elusive. YTHDF2 plays important roles in transcript turnover during animal development [18–20]. We thus hypothesized that YTHDF2 facilitates the timely turnover of phase-specific transcripts to ensure cell cycle progression. To test this, we generated *YTHDF2* knockout HeLa cell lines by using CRISPR-Cas9 (Fig 2A and S3A Fig). We found that all the *YTHDF2* knockout lines showed slower proliferation compared with the wild type (Fig 2B) and that the proliferation defect can be restored by YTHDF2 transfection (S3B Fig). *YTHDF2* knockdown by small interfering RNA (siRNA) displayed consistent slower proliferation (S3C Fig), indicating that YTHDF2 promotes cell proliferation possibly by facilitating mRNA degradation during cell cycle.

To further test this hypothesis, we attempted to obtain a more confident set of YTHDF2-bound transcripts by performing YTHDF2 RNA immunoprecipitation (RIP) using unsynchronized cells followed by sequencing. Based on the YTHDF2 RIP sequencing (RIP-seq) data and the published YTHDF2 photoactivatable ribonucleoside-enhanced crosslinking and immunoprecipitation (PAR-CLIP) data [10], we identified a total of 2,701 common genes as confident YTHDF2 targets (S4A Fig) (S3 Table), 1,975 (73.1%) of which were detected with significant m^6^A enrichment. To compare the expression changes of YTHDF2 target and nontarget genes, wild-type and *YTHDF2* knockout cells were collected for RNA-seq at 0, 2, 4, 6, 8, and 10 hours post release from G_1_/S synchronization. Cumulative fractions of gene expression changes between knockout and wild-type cells showed that the 2,701 YTHDF2 targets displayed significantly higher accumulation from 2 to 10 hours post release in the absence of YTHDF2, compared with the 4,668 nontargets (S4B Fig). These results confirm that YTHDF2 generally plays a role in facilitating mRNA degradation during cell cycle.

A previous study reported a group of genes that express in phase-specific patterns in HeLa cells [36]. We thus examined how these genes are impacted upon YTHDF2 depletion. Among the phase-specific transcripts, 188 are confident YTHDF2 targets (Fig 2C), the majority (80.3%) of which were detected with significant m^6^A enrichment. We found that the 188 phase-specific YTHDF2 targets displayed significantly higher accumulation at 8 and 10 hours post release in the absence of YTHDF2, compared with the 515 nontargets (Fig 2D) (S4 Table). These results suggest that YTHDF2 binds to a subset of phase-specific transcripts to promote their degradation throughout the late G_2_ phase and mitosis.

### YTHDF2 promotes mitotic entry by negatively regulating Wee1-like protein kinase

**Fig 3:**
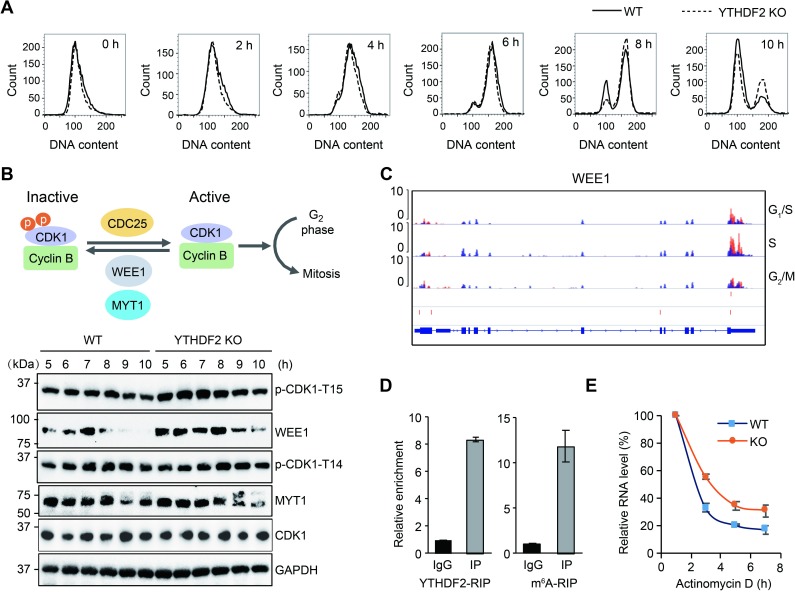
WEE1 is negatively regulated by YTHDF2. (A) Flow cytometry analysis of wild-type and *YTHDF2* knockout cells that were synchronized and released from G_1_/S phase using double thymidine block. (B) Western blot analysis of key proteins that determine mitosis entry. The upper panel illustrates the key regulators of mitotic entry. The lower panel indicates western blot results from wild-type and *YTHDF2* knockout samples that were synchronized to G_1_/S phase using double thymidine block. Samples were collected at times as indicated in the figure post release. (C) Genome browser view of *WEE1* m^6^A peaks at different phases of the cell cycle. The red bars in lower 2 tracks represent the m^6^A sites identified by 2 replicates of YTHDF2 PAR-CLIP data [10]. (D) YTHDF2 and m^6^A RIP-qPCR for *WEE1*. Rabbit IgG was used as control. (E) *WEE1* mRNA decay assay using actinomycin D treatment. The relative levels of *WEE1* transcripts were normalized to the housekeeping gene *HPRT1*. Underlying data for this figure can be found in S1 Data. CDC25, cell division cycle 25; CDK1, cyclin-dependent kinase 1; GAPDH, glyceraldehyde 3-phosphate dehydrogenase; IgG, immunoglobulin G; IP, immunoprecipitation; KO, knockout; m^6^A, *N*^6^-methyladenosine; MYT1, membrane associated tyrosine/threonine 1; p-CDK1-T15, phosphorylating CDK1 at threonine 14; PAR-CLIP, photoactivatable ribonucleoside-enhanced crosslinking and immunoprecipitation; qPCR, quantitative PCR; RIP, RNA immunoprecipitation; WEE1, Wee1-like protein kinase; WT, wild type; YTHDF2, YTH *N*^6^-methyladenosine RNA binding protein 2.

We further investigated whether YTHDF2 generally promotes all the phases of the cell cycle or a specific phase by synchronizing both wild-type and *YTHDF2* knockout lines to G_1_/S phase, followed by cytometry analysis at different time points post release. *YTHDF2* knockout cells show marked delay at 8 hours post release, corresponding to G_2_/M transition (Fig 3A), confirming that YTHDF2 plays a role in promoting mitotic entry.

We next explored the underlying mechanism of mitotic entry regulation mediated by YTHDF2 by assessing protein levels of genes that are important for G_2_/M transition or mitosis from 5 to 10 hours after G_1_/S release by western blots. Mitotic entry is predominantly determined by the phosphorylation status of cyclin-dependent kinase 1 (CDK1), which is controlled by phosphorylation activities of Wee1-like protein kinase (WEE1) and membrane associated tyrosine/threonine 1 (MYT1), and dephosphorylation by cell division cycle 25 (CDC25) [40]. We found that the protein level of CDK1 remained steady across the period in both wild-type and knockout cells (Fig 3B). Interestingly, WEE1—but not MYT1—showed dramatic overaccumulation, suggesting that WEE1 is negatively regulated by YTHDF2 (Fig 3B and S5A Fig). WEE1 and MYT1 are negative regulators of CDK1 by phosphorylating CDK1 at tyrosine 15 (p-CDK1-Y15) and threonine 14 (p-CDK1-T14), respectively [41,42]. Consistent with the higher levels of WEE1 in *YTHDF2* knockout cells, p-CDK1-Y15—but not p-CDK1-T14—is accordingly excessive in the knockout cells (Fig 3B and S5A Fig). RNA-seq data also confirmed higher expression of *WEE1* after 4 hours post synchronization, corresponding to G_2_ to M phase (S5B Fig), consistent with the protein level change (S5C Fig). Moreover, overexpression of WEE1 resulted in slower cell proliferation and higher ratio of cells at G_2_/M phase, suggesting delay of mitotic entry (S5D Fig). These results confirm that YTHDF2 represses the key negative regulator WEE1 from G_2_ to M phase to maintain CDK1 activity.

From the m^6^A MeRIP-seq and PAR-CLIP data, we found that WEE1 transcripts indeed contain m^6^A modification mainly at the 3′ UTR region (Fig 3C), suggesting that WEE1 is regulated at the epitranscriptomic level through YTHDF2. RIP using either m6A or YTHDF2 antibody followed by reverse transcription quantitative PCR (RT-qPCR) confirmed that WEE1 transcript is indeed methylated and bound by YTHDF2 (Fig 3D). RNA decay assay using actinomycin D–mediated transcription inhibition showed that WEE1 transcripts degrade slower in the absence of YTHDF2, demonstrating that YTHDF2 shortens the lifetime of WEE1 transcripts (Fig 3E). Moreover, knockdown of *YTHDF2* or *METTL3* using siRNAs consistently increased both WEE1 mRNA and protein levels, which led to an overaccumulation of p-CDK1-Y15 (S5E Fig), suggesting that m^6^A modification of the WEE1 transcript predominantly promotes its degradation through YTHDF2. Knockdown of YTHDF2 also resulted in higher METTL3 transcript accumulation, but not the protein level (S5E Fig), implicating other mechanisms as regulators of METTL3. Moreover, regardless of the effect on WEE1, knockdown of METTL3 resulted in marked delay from G_1_ to S phase (S5F Fig), suggesting more complex regulatory pathways downstream of METTL3 that regulate cell cycle. Collectively, these results show that YTHDF2 mediates the decay of WEE1 transcripts to ensure timely mitotic entry.

### YTHDF2 stability is maintained by CDK1

**Fig 4:**
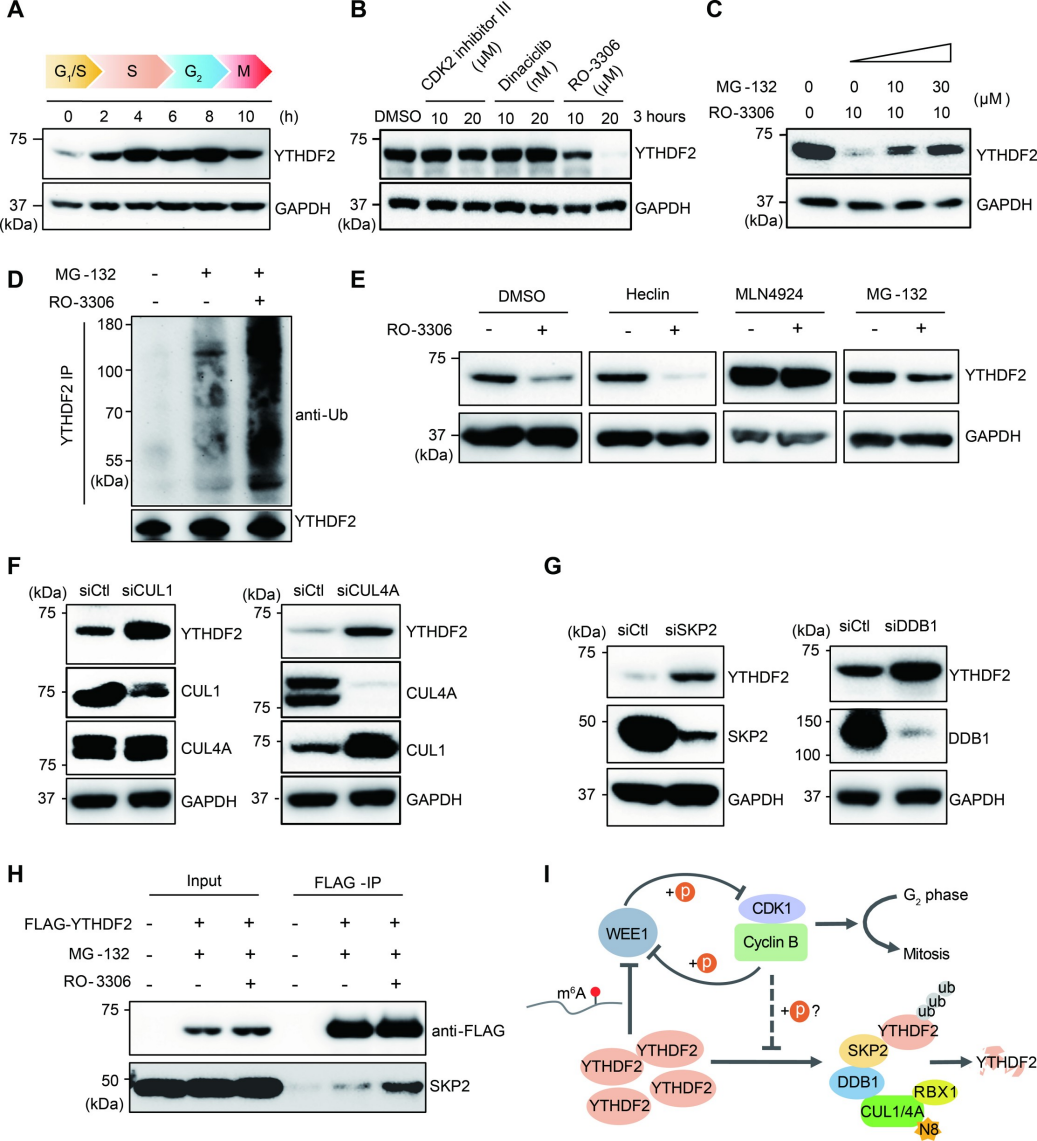
YTHDF2 stability is dependent on CDK1 activity. (A) Western blot of YTHDF2 post G_1_/S synchronization. Samples were collected at the indicated hours post release. (B) Western blot of YTHDF2 using cells treated with small molecule inhibitors. Cells were treated with CDK2 inhibitor III, dinaciclib, and the CDK1 inhibitor RO-3306 in different concentrations as indicated for 3 hours. (C) Western blot of YTHDF2 using cells pretreated with the proteasome inhibitor MG-132 before the CDK1 inhibitor RO-3306 treatment. Cells were incubated with MG-132 with indicated concentrations for 3 hours before RO-3306 treatment. (D) YTHDF2 IP followed by western blot with ubiquitin antibody and YTHDF2 antibody. Cells were pretreated with MG-132 3 hours before being incubated for 3 hours with RO-3306. (E) Small molecular inhibitor assays to identify the proteolysis pathway for YTHDF2. Cells were pretreated with indicated inhibitors 3 hours before treating with RO-3306 for 3 hours for western blots. (F–G) siRNA knockdown of *CUL1*, *CUL4A*, *SKP2*, and *DDB1*, followed by western blot for YTHDF2, CUL1, CUL4A, SKP2, and DDB1. Control siRNA knockdown is indicated as “siCtl.” (H) FLAG-IP using cells sequentially treated with MG-132 and RO-3306 as indicated. Levels of FLAG-YTHDF2 and SKP2 were detected by western blots with anti-FLAG and anti-SKP2 antibodies, respectively. (I) A proposed model of feedforward loop regulating mitosis entry involving CDK1, YTHDF2, and WEE1. WEE1 and CDK1 reciprocally repress each other by phosphorylation to regulate mitotic entry. CDK1 stabilizes YTHDF2 by preventing YTHDF2 from associating with the E3 ligase complexes for degradation. Whether this relies on direct phosphorylation by CDK1 remains to be explored (labeled with question mark). YTHDF2 represses WEE1 by promoting the degradation of WEE1 transcripts via m^6^A. YTHDF2 is degraded after polyubiquitination by the E3 ligase complexes. Underlying data for this figure can be found in S1 Raw Images. anti-Ub, ubiquitin antibody; CDK1, cyclin-dependent kinase 1; CUL1, Cullin 1; DDB1, damaged DNA-binding protein 1; GAPDH, glyceraldehyde 3-phosphate dehydrogenase; IP, immunoprecipitation; m^6^A, *N*^6^-methyladenosine; RBX1, RING-box protein 1; siCtl, control siRNA; siRNA, small interfering RNA; SKP2, S-phase kinase-associated protein 2; WEE1, Wee1-like protein kinase; YTHDF2, YTH *N*^6^-methyladenosine RNA binding protein 2.

We assessed YTHDF2 levels in synchronized cells and found substantially higher accumulation of YTHDF2 protein from late S to G_2_/M phases (Fig 4A), but not the YTHDF2 transcript expression (S6A Fig), indicating that cell cycle controls YTHDF2 at the protein level. To further verify this, we tested a few small molecule inhibitors for cyclin-dependent kinases in cell culture. We found that YTHDF2 is quickly degraded when HeLa cells are incubated with the selective CDK1 inhibitor RO-3306 [43], but not CDK2 Inhibitor III, a selective CDK2 inhibitor [44], or the general kinase inhibitor dinaciclib [45] (Fig 4B). The same effect of RO-3306 was also observed for human embryonic kidney 293T (HEK 293T) cells (S6B Fig), indicating a general effect of CDK1 inhibition on YTHDF2 degradation. To rule out the off-target effect of RO-3306, we tested a different selective CDK1 inhibitor, purvalanol A [46], and found the same effect on YTHDF2 in HeLa cells (S6C Fig). In contrast, any other kinase inhibitors—such as rapamycin and Torin1 for mammalian target of rapamycin (mTOR) [47] and GDC-0994 and SCH772984 for extracellular signal-regulated kinase (ERK) [48]—did not cause YTHDF2 degradation (S6D Fig), suggesting that YTHDF2 degradation is specifically activated by CDK1 inhibition.

Recent studies have shown that loss of YTHDF2 results in enhancement of hematopoietic stem cell expansion in both mouse and human [21,22]. In contrast, depletion of YTHDF2 in AML resulted in apoptosis of AML cells [23]. Thus, YTHDF2 represents a potential selective target for AML treatment. We tested whether CDK1 inhibition also causes YTHDF2 degradation in AMLs to trigger apoptosis. Interestingly, YTHDF2 showed dramatic reduction upon RO-3306 and purvalanol A treatment in both NB4 and MonoMac-6 cells (S6E Fig). Consistently, both AML cells underwent apoptosis along with YTHDF2 degradation, as revealed by 2 apoptosis markers poly (ADP-ribose) polymerase (PARP) and cleaved Caspase-3 (S6E Fig). These results suggest that CDK1 inhibitors promote YTHDF2 degradation in AMLs and might serve as a potential therapeutic method in AML treatment.

The ubiquitin-proteasome pathway mediates the proteolysis of important cell cycle regulators [49]. We hypothesized that YTHDF2 might undergo the same degradation pathway. By pretreating cells with the proteasome inhibitor MG-132, we found that the effect of RO-3306 was antagonized (Fig 4C), suggesting that YTHDF2 proteolysis occurs on proteasomes. Immunoprecipitation of YTHDF2 from cells with sequential MG-132 and RO-3306 treatment revealed a dramatic increase of polyubiquitinated YTHDF2 fraction (Fig 4D), confirming that CDK1 inhibition results in the polyubiquitination of YTHDF2 and subsequent degradation by proteasomes. Taken together, these results show that CDK1, YTHDF2, and WEE1 form a feedforward regulatory loop regulating the onset of mitosis.

### Proteolysis pathway of YTHDF2

We next sought to identify the ubiquitination pathway that is responsible for YTHDF2 proteolysis. Homologous to the E6-AP Carboxyl Terminus (HECT) and Really Interesting New Gene (RING) E3 ligases are among the major classes of ubiquitin ligases [50]. The NEDD8-activating enzyme (NAE) is an E1 enzyme critical for the neddylation pathway that controls the activity of multi-subunit cullin-RING-type E3 ligases (CRLs) [51]. By comparing the effects of the HECT E3 inhibitor Heclin [52] and NAE inhibitor MLN4924 [53], we found that MLN4924—but not Heclin—profoundly antagonized the effect of RO-3306 (Fig 4E). Moreover, treatment of HeLa cells with different concentrations of MLN4924 for 24 hours caused high levels of YTHDF2 accumulation (S7A Fig), similar to p27, which is known to be controlled by NAE [53]. These results suggest that the NAE-mediated neddylation is an essential step for YTHDF2 degradation.

To rule out that RO-3306 treatment possibly activates NAE activity leading to downstream YTHDF2 degradation, we assessed the level of neddylated form of UBC12 (UBC12-NEDD8) catalyzed by NAE. No increase in UBC12-NEDD8 was observed after RO-3306 treatment (S7B Fig). Consistently, although MLN4924 antagonizes the effect of RO-3306 (Fig 4E), RO-3306 does not counteract the effect of MLN4924 in decreasing the conjugated form of UBC12 and NEDD8 (S7C Fig). These results suggest that CDK1 inhibition activates YTHDF2 degradation downstream of E1 and E2 ligases in the CRL pathway. In addition, the reduction of UBC12-NEDD8 conjugation induced by MLN4924 indicates that RING-box protein 1 (RBX1)—but not RBX2—participates in the YTHDF2 degradation pathway because UBC12 and RBX1 are specific pairs in the CRL pathway [54].

In order to identify the CRL components, we performed siRNA-mediated knockdown of a few cullins, including *CUL1*, *CUL4A*, *CUL4B*, and *CUL7*. Both *CUL1* and *CUL4A* knockdown, but not *CUL4B* and *CUL7*, led to the overaccumulation of YTHDF2 (Fig 4F and S7D Fig), suggesting that YTHDF2 degradation is mediated by distinct E3 ligase complexes. Cullin 1 (CUL1) primarily forms E3 ligase complexes with S-phase kinase-associated protein 1 (SKP1) and SKP2, mediating the degradation of many important cell cycle regulators [55,56]. However, knockdown of *SKP1* did not increase YTHDF2 accumulation (S7E Fig), suggesting that CUL1 might form complexes with alternative adaptors rather than SKP1 to mediate YTHDF2 degradation. Unexpectedly, we found that *CUL4A* knockdown led to the overaccumulation of CUL1 (Fig 4F), implicating a hierarchical cascade of CRLs, in which CUL4A controls the proteolysis of CUL1.

Damaged DNA-binding protein 1 (DDB1) is a known adaptor of CUL4A [57] and might interact with substrate receptor SKP2 [58] or CDT2 [59]. Knockdown of *DDB1* and *SKP2*, but not *CDT2*, caused dramatic YTHDF2 increase (Fig 4G and S7F Fig), suggesting that CUL4A, DDB1, and SKP2 might form complexes for YTHDF2 degradation. To further verify this, a stable HeLa cell line expressing FLAG-YTHDF2 was sequentially treated with MG-132 and RO-3306. Immunoprecipitation using anti-FLAG M2 beads revealed that a higher fraction of SKP2 was co-immunoprecipitated with FLAG-YTHDF2 when CDK1 was inhibited by RO-3306 (Fig 4H). These results confirm that SKP2 is the substrate receptor of YTHDF2 and that CDK1 inhibition increases the association between YTHDF2 and SKP2.

## Discussion

Transcriptomic m^6^A participates in a variety of biological processes, such as cell differentiation, development, memory, and immunity. Although it has been implicated in cell cycle regulation, systematic and mechanistic studies are still lacking. In this study, we investigated whether transcriptomic m^6^A is impacted by cell cycle regulation and found that m^6^A-modified transcripts, especially the ones with phase-specific expression patterns, display dynamic changes during cell cycle. It has been reported that mRNA synthesis and decay show dynamic changes during yeast cell cycle [39]; however, factors that contribute to the fast turnover of mRNAs remain elusive. This study revealed that YTHDF2 regulates a subset of phase-specific genes, confirming the regulatory role of transcriptomic m^6^A modification in promoting RNA degradation during cell cycle.

Further investigation found that YTHDF2 depletion leads to delayed mitotic entry. By meticulously comparing a number of key regulators gating mitotic entry, we found that WEE1, a suppressor of CDK1 by phosphorylation, is regulated by YTHDF2 by promoting WEE1 transcript decay. Therefore, overaccumulation of WEE1 protein upon YTHDF2 depletion leads to the delay of G_2_ to M phase transition by increasing the ratio of the inactive CDK1 form—CDK1-Y15. Phosphorylation and dephosphorylation of regulators, such as CDK1 and WEE1, are important mechanisms that regulate mitosis [40,60,61]. Here, we reveal that, in addition to the regulation at the protein level, the m^6^A modification of cell cycle regulator transcripts represents another pathway to fine-tune cell cycle progression. Future epitranscriptomic studies of important cell cycle regulators in different cell types and biological processes could provide more insights.

By testing a number of small molecule inhibitors, we found that the YTHDF2 protein stability relies on CDK1 activity. YTHDF2 undergoes dramatic polyubiquitination and subsequent degradation upon CDK1 inhibition. This result indicates that CDK1 maintains YTHDF2 stability during cell cycle, which represses WEE1 at the RNA level via m^6^A. However, whether CDK1 maintains YTHDF2 stability by directly phosphorylating YTHDF2 or through an indirect mechanism remains to be investigated in the future. Considering that CDK1 represses WEE1 by phosphorylation at serine 123 [62], CDK1, YTHDF2, and WEE1 thus form a feedforward regulatory loop that regulates mitotic entry (Fig 4I).

Adopting a similar chemical biology approach, we interrogated the degradation pathway of YTHDF2. We found that YTHDF2 proteolysis occurs on proteasomes mediated by the CRL-NEDD8 pathway, which is known for regulating many cellular processes, including cell cycle progression [49]. Screening for individual components of the potential E3 ligases using siRNAs identified CUL1, CUL4A, DDB1, and SKP2 as responsible for YTHDF2 proteolysis. We found that CDK1 maintains YTHDF2 stability likely by preventing the association between YTHDF2 and the SKP2-involved E3 ligase complexes. Moreover, our results suggested that CUL1 proteolysis is dependent on CUL4A. However, overaccumulation of CUL1 is not sufficient to compensate for YTHDF2 degradation in the absence of CUL4A, suggesting that CUL4A and CUL1 may function in a cascading yet distinct manner in mediating protein degradation.

In summary, we revealed a novel regulatory mechanism involving m^6^A that serves as an additional level of regulation in cell cycle control, in addition to the well-known regulations through protein phosphorylation and degradation. Moreover, we identified a feedforward regulatory loop that consists of CDK1, YTHDF2, and WEE1 regulating mitotic entry. The recent study revealed that YTHDF2 is essential for AML cells and depletion of YTHDF2 leads to the apoptosis of AML cells [23], rendering YTHDF2 a potential target for AML treatment. Our results highlight the potential of CDK1 inhibitors in inducing YTHDF2 degradation and apoptosis in AMLs. Moreover, the discovery of the YTHDF2 proteolysis pathway could lay a foundation for developing additional methods to manipulate YTHDF2 levels by techniques such as proteolysis targeting chimera (PROTAC) [63]. Therefore, elucidation of how YTHDF2 is regulated and its proteolysis pathway would potentially benefit the development of new therapeutic strategies to manipulate YTHDF2 levels in cancers—such as AML [23]—or for ex vivo amplification of hematopoietic stem cells [21,22].

## Materials and methods

### Cell culture

Human HeLa and HEK 293T cell lines were purchased from ATCC and maintained in DMEM (Gibco) with 10% fetal bovine serum (FBS) (Gibco) and 1% penicillin/streptomycin (Gibco) in a humidified 37°C incubator with 5% CO_2_. NB4 cells were grown in RPMI medium 1640 (Gibco) with 10% FBS, 1% HEPES (Gibco), and 1% penicillin/streptomycin. MonoMac-6 cells were grown in RPMI 1640 supplemented with 10% FBS, 1% HEPES, 2 mM L-Glutamine (Gibco), 1% nonessential amino acids solution (Gibco), 1 mM sodium pyruvate (Gibco), 9 μg/ml insulin (MilliporeSigma), and 1% penicillin/streptomycin.

### Plasmid construction

Human full-length YTHDF2 cDNA was cloned and inserted into the PiggyBac vector with a FLAG tag at the N-terminus. High-purity plasmids used for mammalian cell transfection and stable cell line preparation were prepared using the HiSpeed Plasmid Midi Kit (Qiagen). The WEE1 construct (Category Number RC209760) and empty vector control (Category Number PS100001) were ordered from OriGene.

### siRNA knockdown and plasmid transfection

Cells were resuspended 16 hours prior to both siRNA and plasmid transfections. siRNAs were transfected to cells at approximately 50% confluency using the transfection reagent Lipofectamine RNAiMAX (Invitrogen). Cells reaching approximately 70% confluency were transfected with plasmids using Lipofectamine 2000 (Invitrogen). Both siRNA and plasmid transfections were conducted following the manufacturer’s protocols. Transfected cells were harvested 48 hours post siRNA or plasmid transfections for the following experiments. For stable FLAG-YTHDF2 HeLa cell line generation, DMEM medium supplemented with 1 μg/mL puromycin was used for selection 48 hours post plasmid transfection. Cells were maintained in medium with 1 μg/mL puromycin for at least a week before experiments.

### CRISPR-Cas9–mediated knockout

YTHDF2 knockout was performed using the Alt-R CRISPR-Cas9 System from Integrated DNA Technologies (IDT) in HeLa cells. Guide RNA sequences were designed using CHOPCHOP [64] and synthesized as crRNAs from IDT (S5 Table). Both Alt-R CRISPR-Cas9 tracrRNA and synthesized crRNAs were dissolved to 100 μM in nuclease-free IDTE buffer (IDT), while Alt-R S.p. Cas9 Nuclease V3 (IDT) was diluted to 1 μM in Cas9 working buffer (20 mM HEPES [pH 7.5], 150 mM KCI). Equimolar tracrRNA and crRNA were mixed to reach a final concentration of 1 μM of each oligo in nuclease-free duplex buffer (IDT) and annealed by heating at 95°C for 5 minutes and cooling down to room temperature. Annealed oligos (1.5 μL), diluted Cas9 enzyme (1.5 μL), and 22 μL Opti-MEM (Gibco) were mixed and incubated at room temperature for 5 minutes to assemble the RNP complexes. After the incubation, 1.2 μL of Lipofectamine RNAiMAX and 23.8 μL Opti-MEM were added to the RNP complex solution and incubated for 20 minutes at room temperature. The transfection complexes were then added to a 96-well plate containing 40,000 cells/100 μL suspensions per well, followed by incubation at 37°C for 48 hours. Single-cell colonies were obtained by serial dilution of cells harvested from each well. Primers flanking the guide RNA sites were synthesized to amplify the DNA from the knockout cell lines derived from single colonies. PCR products were cloned to plasmids using the TOPO TA Cloning Kit (Invitrogen) for Sanger sequencing, which confirmed that CRISPR mostly caused open reading frame shifts, with at least one copy of the *YTHDF2* gene in line KO-4 having 39-bp deletion, resulting in the deletion of 13 amino acid residues.

### Cell proliferation assay

Cells were suspended in medium and counted using the Countess Automated Cell Counter (Invitrogen). About 3,000 cells were seeded to each well of the 96-well plates. After the cells adhered to the plate, 10 μL of the Cell Counting Kit-8 (MilliporeSigma) solution was added to each well and incubated for 2 hours in the 37°C CO_2_ incubator, followed by measuring of the absorbance at 450 nm using the Synergy HTX Multi-Mode Microplate Reader (BioTek). Absorbance was measured for 3 additional days to calculate the relative cell proliferation. To measure the proliferation of cells with siRNA knockdown, cells were transfected with siRNAs using Lipofectamine RNAiMax (Invitrogen) at least 6 hours before trypsinization for cell counting and seeding into 96-well plates. At least 5 wells were measured to calculate the average absorbance for each cell line at each time point.

### Cell synchronization

HeLa cells were synchronized to early S phase using the double thymidine block based on a previous protocol [65]. Briefly, HeLa cells were grown in 10 cm plates with 10 mL medium to reach approximately 40% confluency, and then 200 uL of 100 mM thymidine stock solution was added to the cell culture to a final concentration of 2 mM. Cells were incubated in the 37°C CO_2_ incubator for 14 hours and then washed with 10 mL PBS twice. Growth medium supplemented with 24 μM deoxycytidine was added to the cells and incubated for 9 hours. The amount of 200 μL of 100 mM thymidine stock solution was added to the cell culture and incubated for 14 hours. To release the block, cells were washed with 10 mL PBS twice, and 10 mL medium was added for cell cycle progression. Cells were collected at different time points for up to 10 hours for experiments.

### Flow cytometry

Single-cell suspensions were acquired by treating cells with trypsin solution at different time points post double thymidine block and mixed with equal volume of freshly prepared 4% paraformaldehyde (PFA) solution in PBS to reach a final concentration of 2% PFA. Cells were fixed at room temperature for 15 minutes, followed by washing twice with PBS. Cells were stained in 5 μg/mL Hoechst 33342 (MilliporeSigma) in PBS for 15 minutes at 37°C before flow cytometry. Ultraviolet light with 355-nm wavelength and 450/50-nm bandpass filters were used to quantify the DNA content of the cells for flow cytometry.

### Small molecule inhibitor treatment

Small molecule inhibitors were dissolved in DMSO as stock solutions. Inhibitors were added to cell cultures with final concentrations and incubation times as indicated in each figure legend. For proteasome or E3 ligase inhibition assays, cells were incubated with 10 μM MG-132 (Selleckchem) [66], 100 μM Heclin (MilliporeSigma) [67], or 5 μM MLN4924 (MilliporeSigma) [53] for 3 hours, followed by 10 μM RO-3306 (MilliporeSigma) [66] before collecting the cells for western blots or protein co-immunoprecipitations. The concentrations of CDK2 inhibitor III, dinaciclib, purvalanol A, mTOR inhibitors, and ERK inhibitors were determined by the manufacturer’s instructions or previous reports [44,45,68–70]. For transcription inhibition assays, a final concentration of 5 μg/mL actinomycin D (MilliporeSigma) [10] was added to cell cultures at 6 hours, 3 hours, and 0 hours before cell collection for RNA extraction.

### Protein co-immunoprecipitation and western blot

A stable line of HeLa cells expressing FLAG-YTHDF2 was collected from 15 cm plates and pelleted by centrifuge at 400*g* for 5 minutes. Cell pellets were resuspended with 3 volumes of immunoprecipitation lysis buffer (25 mM Tris-HCl [pH 7.4], 150 mM NaCl, 1 mM EDTA, 1% NP-40, and 5% glycerol, 1:50 protease inhibitor cocktail) and incubated on ice for 20 minutes. Cell lysates were then centrifuged at 16,000*g* for 15 minutes at 4°C. We saved 50 μl of cell lysate as input and incubated the rest with pre-equilibrated 50 μl anti-FLAG M2 magnetic beads (MilliporeSigma) by rotating at 4°C for 2 hours. After incubation, beads were subjected to washing 6 times with lysis buffer and boiled in NuPAGE LDS Sample Buffer (Invitrogen) with 5% 2-mercaptoethanol (2-ME). After magnetic separation, the supernatant was transferred as immunoprecipitation sample for western blotting, together with 1% input.

For samples collected for western blot, cells were lysed using RIPA buffer (Invitrogen) containing 1:100 protease inhibitor cocktail (Invitrogen) and phosphatase inhibitor cocktail (Invitrogen) on ice for 20 minutes. After centrifuging at 16,000*g* for 15 minutes at 4°C, the supernatant was transferred and boiled in NuPAGE LDS Sample Buffer (Invitrogen) with 5% 2-ME (MilliporeSigma). Samples were loaded to a NuPAGE 4% to 12% Bis-Tris Protein Gel (Invitrogen) for electrophoresis at a constant voltage of 160 V until the front dye reached the bottom of the gel and were transferred to 0.2 or 0.45 μM nitrocellulose membranes (BioRad) using the Trans-Blot SD semi-dry transfer cell (BioRad), followed by blocking with 5% nonfat milk (BioRad) in TBST. Antibody concentrations used for western blots were adjusted based on the manufacturer’s instructions.

### RNA purification

Total RNA was purified from cells with the TRIzol reagent (Thermo Scientific). Polyadenylated RNA was purified from total RNA using the Dynabeads mRNA DIRECT kit (Thermo Scientific) according to the manufacturer’s protocol. mRNA concentrations were measured by Qubit fluorometer (Invitrogen) with the Qubit RNA HS Assay Kit (Invitrogen).

### LC-MS/MS

The amount of 300 ng of isolated polyadenylated RNA was further processed using RiboMinus Transcriptome Isolation Kit (Invitrogen). The eluted RNA was then digested by nuclease P1 (MilliporeSigma) followed by dephosphorylation by alkaline phosphatase (Invitrogen). The samples were then used for LC-MS/MS. For detailed procedures for sample process and mass spectrometry, refer to the previous report [10].

### RNA-seq

Total RNA was extracted from HeLa cells at different time points post release from double thymidine block and was subjected to polyadenylated RNA isolation using the Dynabeads mRNA DIRECT kit (Thermo Scientific). The amount of 100 ng of mRNA samples was used for RNA-seq library construction using the TruSeq stranded mRNA sample preparation kit (Illumina) by following the manufacturer’s protocol. High-throughput libraries for 2 biological replicates were sequenced using Illumina HiSeq 4000 with single-end 50-bp read length at The University of Chicago.

### RIP-seq

Unsynchronized HeLa cells with 90% confluency in 15 cm plates were collected by cell lifters, pelleted by centrifuge for 5 minutes at 400*g*, and washed once with cold PBS. The cell pellets were lysed with 3 volumes of lysis buffer (150 mM KCl, 10 mM HEPES [pH 7.6], 2 mM EDTA, 0.5% NP-40, 0.5 mM DTT, 1:50 protease inhibitor cocktail, 1 U/μL SUPERase•In RNase Inhibitor) on ice for 20 minutes. The lysates were centrifuged at 16,000*g* for 15 minutes; 50 μL cell lysate was saved as input and mixed with 1 mL TRIzol for RNA extraction; and 20 μg of YTHDF2 antibody was added to the rest of the lysate and incubated by rotating for 2 hours at 4°C. Afterward, 40 μL of Pierce Protein A/G Magnetic Beads was washed twice with lysis buffer and added to the lysate and was incubated by rotating for 1 hour at 4°C. After separation using a magnetic rack, beads were washed with ice-code lysis 6 times. Beads were then mixed with 1 mL TRIzol and recovered for RNA, which was saved as immunoprecipitation. RNA extracted from input was further subjected to mRNA purification using the Dynabeads mRNA DIRECT kit (Thermo Scientific). Input mRNA of 100 ng and immunoprecipitation RNA samples with 2 biological replicates were used for RNA-seq library preparation.

### RT-qPCR

For reverse transcription, 100 ng of total RNA or RIP extracted using TRIzol was subjected to DNA depletion using the TURBO DNA-free kit (Invitrogen) according to the manufacturer’'s protocol. The resultant DNA-free RNA was reverse-transcribed using the PrimeScript RT Reagent Kit (Takara, Category Number RR037A). The cDNA products were then used for real-time qPCR on the LightCycler 96 System (Roche) using FastStart Essential DNA Green Master (Roche). Gene-specific and housekeeping gene *HPRT1* primers were designed and synthesized from IDT (S5 Table). qPCR analysis was performed using the ΔΔCt method. Average values from 3 replicates were calculated for each sample.

### m^6^A MeRIP-seq

The m^6^A MeRIP-seq library preparation was adapted from the previous published protocol [71]. Briefly, total RNA extracted from cells synchronized at different phases of the cell cycle was subjected to polyadenylated RNA isolation, as described earlier; 5 μg of isolated poly(A)+ RNA was fragmented to approximately 100 nt by sonication using Bioruptor (Diagenode) with 4°C water bath. A portion (100 ng) of the fragmented RNA was then saved as input. The rest of the fragmented RNA was then subjected to m^6^A immunoprecipitation using 15 μg anti-m^6^A antibody (Synaptic Systems) in immunoprecipitation buffer (10 mM Tris-HCl [pH 7.4], 150 mM NaCl, 0.1% NP-40, 1 U/μL SUPERase•In RNase Inhibitor) for 2 hours at 4°C. The amount of 50 μl pre-washed protein A/G beads was added and incubate by rotating for 2 hours, followed by washing 3 times using immunoprecipitation buffer. The m^6^A-containing RNA fragments from beads were eluted twice using 100 μl elution buffer (10 mM Tris-HCl [pH 7.4], 150 mM NaCl, 6.7 mM m^6^A, 0.1% NP-40, 1 U/μL SUPERase•In RNase Inhibitor) for 1 hour while rotating at 4°C. Eluted RNA was then precipitated in 0.3 M sodium acetate (pH 5.2) and 75% ethanol with GlycoBlue (Invitrogen) at −80°C overnight. RNA was recovered from the pellet by centrifuge at 16,000*g* for 20 minutes at 4°C. Both input and immunoprecipitation RNA samples were subjected to RNA-seq library construction.

### Sequencing data analysis

The quality control of sequencing reads of RNA-seq, RIP-seq, and m^6^A-seq libraries was performed using FastQC version 0.11.5. After trimming adapter using Cutadapt version 1.1.5 [72], we then mapped to the human genome GRCh37/hg19 using TopHat version 2.1.0, allowing for at most 2 mismatches [73]. For RNA-seq data, cuffnorm from the Cufflinks (version 2.2.1) package was used for geometric normalization and FPKM calculation [74]. For RIP-seq data analysis, Cufflinks—combined with Cuffdiff—was employed to quantify gene expression and differential analysis. Genes with q < 0.05 were considered YTHDF2 targets. More confident YTHDF2 targets were obtained by intersecting targets from RIP-seq of this study and the published YTHDF2 PAR-CLIP data [10].

For m^6^A-seq data analysis, the m^6^A-enriched regions in each m^6^A-immunoprecipitation sample were identified by MACS2 version 2.1.0 with the option of “—nomodel” [75], using corresponding input library as control. Peaks identified with q < 0.05 were used for downstream analysis. Peak annotation and motif search were both accomplished using the software HOMER [76]. Metagene analysis of m^6^A distribution on transcripts was performed using the MetaPlotR pipeline [77]. IGVtools was used to convert bam files to tdf files, which were loaded into the Integrative Genomics Viewer (IGV) for m^6^A peak visualization [78]. The R package “DiffBind” was used for m^6^A peak differential analysis with a cutoff of *P* < 1 × 10^−3^. Genes with differential m^6^A enrichment at different phases of the cell cycle were subjected to GO analysis using DAVID [79]. GO terms were further summarized and visualized using REVIGO [80].

## Supporting information

S1 Fig m^6^A MeRIP-seq for cells synchronized at G_1_/S, S, and G_2_/M phases. (A) Workflow of cell synchronization by double thymidine block and time points for cell collection after release. (B) GO terms of common genes with m^6^A modifications across 3 phases of the cell cycle. Color key represents the −log(*P* value) of enriched GO terms. (C) Correlation heatmap representing pairwise comparison of m^6^A enrichment for each replicate at 3 phases. (D) Western blot of METTL3, METTL14, and FTO at different time points post synchronization. (E) Expression levels of METTL3, METTL14, and FTO at different time points post synchronization from the RNA-seq data. Underlying data for this figure can be found in S1 Raw Images and S1 Data.
(TIF)

S2 Fig GO terms analysis of genes with differential m^6^A enrichment. (A) Correlation analysis of gene expression levels and m^6^A peak enrichment. (B) Cumulative distribution of gene expression changes with differential m^6^A enrichment at different phases. Left panel shows cumulative distribution by comparing the expression level of transcripts with up- or down-regulated m^6^A from G_1_/S to S phase. Right panel shows that from S to G_2_/M phase. The x-axes indicate the log2 fold change of gene expression level in the next phase compared with the previous phase. *P* values were calculated using the Mann-Whitney test. (C) GO terms for increased m^6^A peaks at S phase compared with G_1_/S. (D) GO terms for decreased m^6^A peaks at S phase compared with G_1_/S. (E) GO terms for increased m^6^A peaks at G_2_/M phase compared with S. TUBB4B is an example that is related to “microtubule-based process” with higher m^6^A at G_2_/M phase. (F) GO terms for decreased m^6^A peaks at G_2_/M phase compared with S. SMAD3 is an example that is related to regulation of transcription with reduced m^6^A from S phase to G_2_/M phase. Underlying data for this figure can be found in S1 Data.
(TIF)

S3 Fig Depletion of YTHDF2 decreases cell proliferation. (A) Design of crRNAs for CRISPR-Cas9 for *YTHDF2* knockout. (B) Rescue of YTHDF2 knockout cell lines by FLAG-YTHDF2 transfection. Two knockout cell lines KO-1 and KO-2 were randomly selected for transfection and proliferation assay. (C) Cell proliferation assays for HeLa cells with *YTHDF2* siRNA knockdown compared with the siRNA control. Underlying data for this figure can be found in S1 Data. crRNA, CRISPR RNA.
(TIF)

S4 Fig Expression and m^6^A changes of genes at different phases of the cell cycle. (A) Intersection for a confident YTHDF2 targets in HeLa cells between YTHDF2 RIP-seq and PAR-CLIP data. PAR-CLIP results are from Wang and colleagues [10]. The 4,668 nontarget genes were obtained after filtering out the genes in either RIP-seq or PAR-CLIP list and the ones with FPKM < 1 in the input sample of the RIP-seq data. (B) Cumulative distribution of 2,701 YTHDF2 targets and 4,668 nontargets by comparing WT and knockout cell lines. Genes with FPKM < 1 at each time point were further removed from the analysis. x-Axes indicate the log2 fold change of gene expression in knockout versus wild type. *P* values were calculated using the Mann-Whitney test. Underlying data for this figure can be found in S1 Data. FPKM, Fragments Per Kilobase of transcript per Million mapped reads.
(TIF)

S5 Fig Cell cycle changes upon YTHDF2 or METTL3 depletion. (A) Quantification of WEE1 and p-CDK1-Y15 by ImageJ from Fig 3B. The protein levels were normalized to the loading control GAPDH. (B) Expression level of *WEE1* revealed by RNA-seq in wild-type and knockout cells at different time points post release from G_1_/S phase. (C) Western blot of WEE1 at different time points post synchronization in wild-type and *YTHDF2* knockout HeLa cells. The right panel shows the normalized values of WEE1 quantified by ImageJ. (D) Effect of WEE1 overexpression in HeLa cells. Left panel shows cell proliferation of HeLa cells transfected with Myc-WEE1 compared with the empty vector control. The right panel shows flow cytometry analysis results of each phase during cell cycle. The percentages of each phase were quantified using FlowJo. (E) siRNA knockdown of *YTHDF2* and *METTL3* in HeLa cells. The left panel shows RT-qPCR results with two-sided Student *t* test (*P < 0.05; **P < 0.01; ***P < 0.001). The right panel shows western blot results of each protein. (F) Flow cytometry results of each phase in the cell cycle upon YTHDF2 or METTL3 knockdown. Underlying data for this figure can be found in S1 Data and S1 Raw Images.
(TIF)

S6 Fig Effects of small molecule inhibitors on YTHDF2 protein stability. (A) The level of YTHDF2 at different time points post synchronization. The black line indicates protein level changes of YTHDF2 quantified by ImageJ. The red dots indicate transcript levels of *YTHDF2* at each time point, which were normalized to the value at 0 hours. (B) RO-3306 induces YTHDF2 degradation in HEK 393T cells within 3 hours. The concentrations of RO-3306 are indicated. (C) Purvalanol A, a different CDK1 inhibitor, induces YTHDF2 degradation. Concentrations and incubation times are as indicated. (D) Detection of YTHDF2 levels after the treatment of relevant inhibitors, including mTOR and ERK inhibitors. (E) Western blot of YTHDF2 and apoptosis markers PARP and Caspase-3 (cleaved form) after CDK1 inhibitor treatment in NB4 and MonoMac-6 cells. Underlying data for this figure can be found in S1 Data and S1 Raw Images.
(TIF)

S7 Fig Identification of the pathway that mediates YTHDF2 degradation. (A) Western blot of YTHDF2 after cells incubated with different concentrations of MLN4924 for 24 hours. p27 is a positive control. (B) Detection of the conjugated form of UBC12-NEDD8 after sequential treatment of RO-3306 and MLN4924. Cells were pretreated with RO-3306 for 3 hours before MLN4924 incubation for 3 hours, with concentrations indicated in the figure. (C) Detection of the conjugated form of UBC12-NEDD8 after treating cells with 10 μM RO-3306 for indicated hours. (D–F) Western blot of YTHDF2 after siRNA knockdown of *CUL4B*, *CUL7*, *SKP1*, and *CDT2*. Underlying data for this figure can be found in S1 Raw Images.
(TIF)

S1 Table m6A peaks at different phases of the cell cycle. (XLSX)

S2 Table Differential analysis of peak enrichment. (XLSX)

S3 Table High-confidence YTHDF2 target genes. (XLSX)

S4 Table Expression levels of phase-specific genes. (XLSX)

S5 Table Oligos and reagents used in this study. (XLSX)

S1 Data Numerical data for figures. (XLSX)

S1 Raw Images Raw images for blots. (PDF)

## Abbreviations

2-ME: 2-mercaptoethanol
ALKBH5: AlkB Homolog 5
AML: acute myeloid leukemia
CCR4-NOT: Carbon Catabolite Repressor 4-Negative on TATA
CDC25: cell division cycle 25
CDK1: cyclin-dependent kinase 1
CRL: cullin-RING-type E3 ligase
CUL1: Cullin 1
CUL4A: Cullin 4A
DDB1: damaged DNA-binding protein 1
ERK: extracellular signal-regulated kinase
FBS: fetal bovine serum
FOXM1: forkhead box protein M1
FTO: fat mass and obesity-associated protein
GO: gene ontology
HECT: Homologous to the E6-AP Carboxyl Terminus
HEK 293T: human embryonic kidney 293T
IDT: Integrated DNA Technologies
IGV: Integrative Genomics Viewer
m^6^A: *N*^6^-methyladenosine
MeRIP-seq: methylated RNA immunoprecipitation sequencing
mESC: mouse embryonic stem cell
METTL3: Methyltransferase Like 3
MRP: Mitochondrial RNA Processing
mTOR: mammalian target of rapamycin
MYT1: membrane associated tyrosine/threonine 1
MZT: maternal to zygotic transition
NAE: NEDD8-activating enzyme
PAR-CLIP: photoactivatable ribonucleoside-enhanced crosslinking and immunoprecipitation
PARP: poly (ADP-ribose) polymerase
p-CDK1-T14: phosphorylating CDK1 at threonine 14
p-CDK1-Y15: phosphorylating CDK1 at tyrosine 15
PFA: paraformaldehyde
PROTAC: proteolysis targeting chimera
RBX1: RING-box protein 1
RING: Really Interesting New Gene
RIP: RNA immunoprecipitation
RIP-seq: RIP sequencing
RNA-seq: RNA sequencing
RT-qPCR: reverse transcription quantitative PCR
siRNA: small interfering RNA
SKP2: S-phase kinase-associated protein 2
UTR: untranslated region
VIRMA: Vir Like m6A Methyltransferase Associated
WEE1: Wee1-like protein kinase
WTAP: Wilms tumor 1-associated protein
YTHDF2: YTH *N*^6^-methyladenosine RNA binding protein 2
ZC3H13: Zinc Finger CCCH-Type Containing 13
