# Supplementary figures and images for: YTHDF2 promotes mitotic entry and is regulated by cell cycle mediators
Source: PLoS Biol. 2020 Apr 8;18(4):e3000664. doi: 10.1371/journal.pbio.3000664 (PMC7170294; doi:10.1371/journal.pbio.3000664)

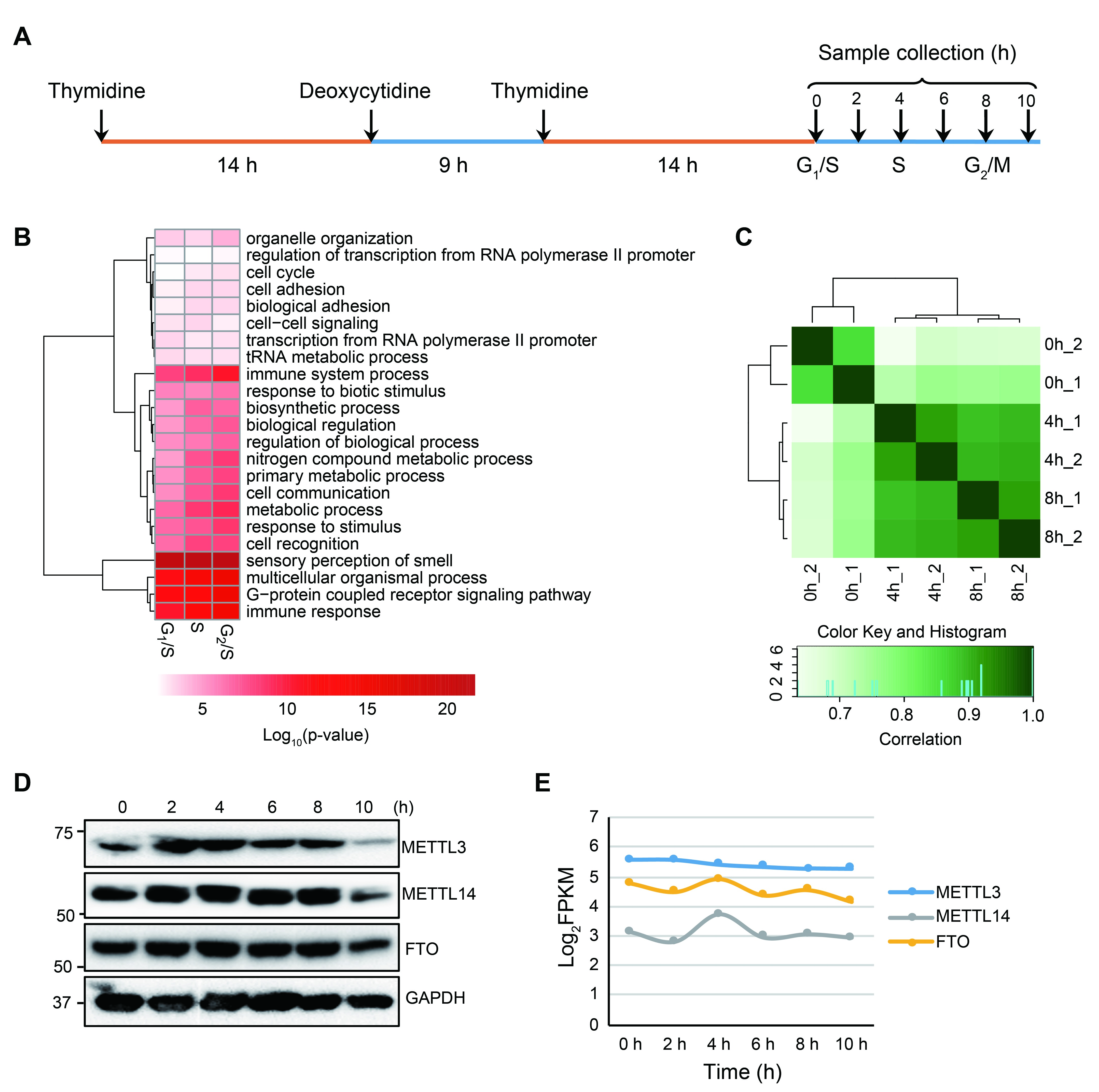

Supplement: S1 Fig — (A) Workflow of cell synchronization by double thymidine block and time points for cell collection after release. (B) GO terms of common genes with m6A modifications across 3 phases of the cell cycle. Color key represents the −log(P value) of enriched GO terms. (C) Correlation heatmap representing pairwise comparison of m6A enrichment for each replicate at 3 phases. (D) Western blot of METTL3, METTL14, and FTO at different time points post synchronization. (E) Expression levels of METTL3, METTL14, and FTO at different time points post synchronization from the RNA-seq data. Underlying data for this figure can be found in S1 Raw Images and S1 Data. (TIF) [file pbio.3000664.s001.tif]

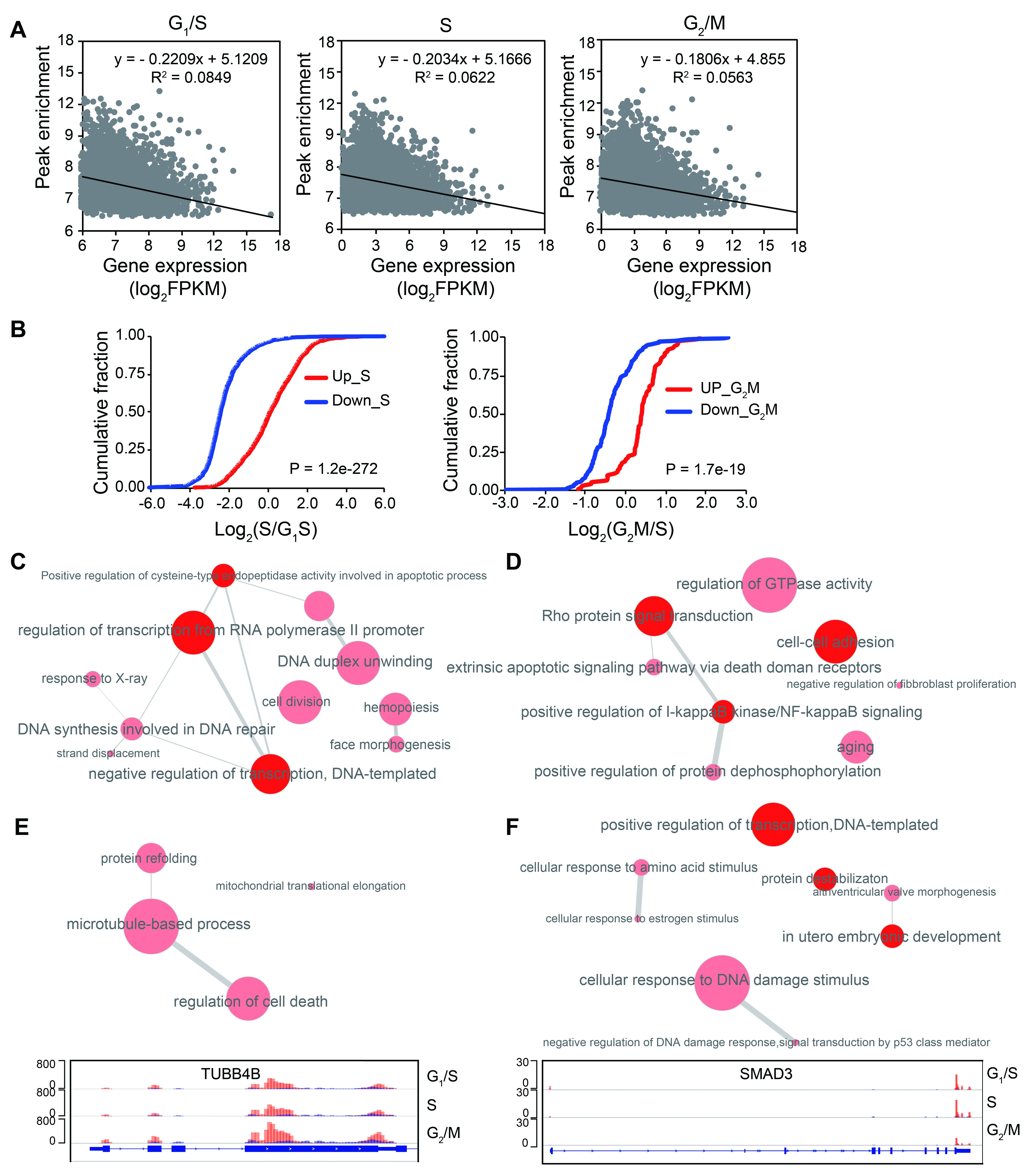

Supplement: S2 Fig — (A) Correlation analysis of gene expression levels and m6A peak enrichment. (B) Cumulative distribution of gene expression changes with differential m6A enrichment at different phases. Left panel shows cumulative distribution by comparing the expression level of transcripts with up- or down-regulated m6A from G1/S to S phase. Right panel shows that from S to G2/M phase. The x-axes indicate the log2 fold change of gene expression level in the next phase compared with the previous phase. P values were calculated using the Mann-Whitney test. (C) GO terms for increased m6A peaks at S phase compared with G1/S. (D) GO terms for decreased m6A peaks at S phase compared with G1/S. (E) GO terms for increased m6A peaks at G2/M phase compared with S. TUBB4B is an example that is related to “microtubule-based process” with higher m6A at G2/M phase. (F) GO terms for decreased m6A peaks at G2/M phase compared with S. SMAD3 is an example that is related to regulation of transcription with reduced m6A from S phase to G2/M phase. Underlying data for this figure can be found in S1 Data. (TIF) [file pbio.3000664.s002.tif]

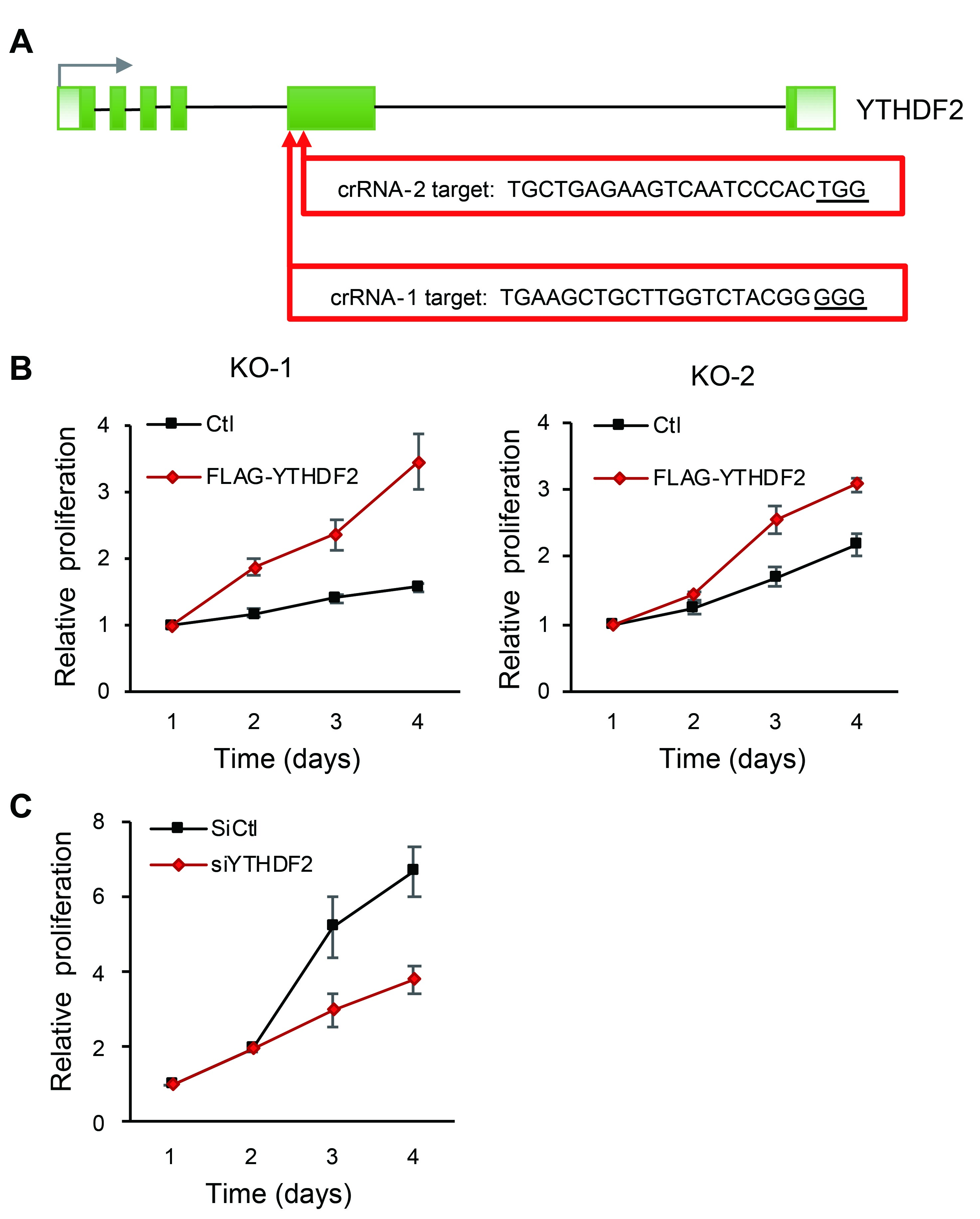

Supplement: S3 Fig — (A) Design of crRNAs for CRISPR-Cas9 for YTHDF2 knockout. (B) Rescue of YTHDF2 knockout cell lines by FLAG-YTHDF2 transfection. Two knockout cell lines KO-1 and KO-2 were randomly selected for transfection and proliferation assay. (C) Cell proliferation assays for HeLa cells with YTHDF2 siRNA knockdown compared with the siRNA control. Underlying data for this figure can be found in S1 Data. crRNA, CRISPR RNA. (TIF) [file pbio.3000664.s003.tif]

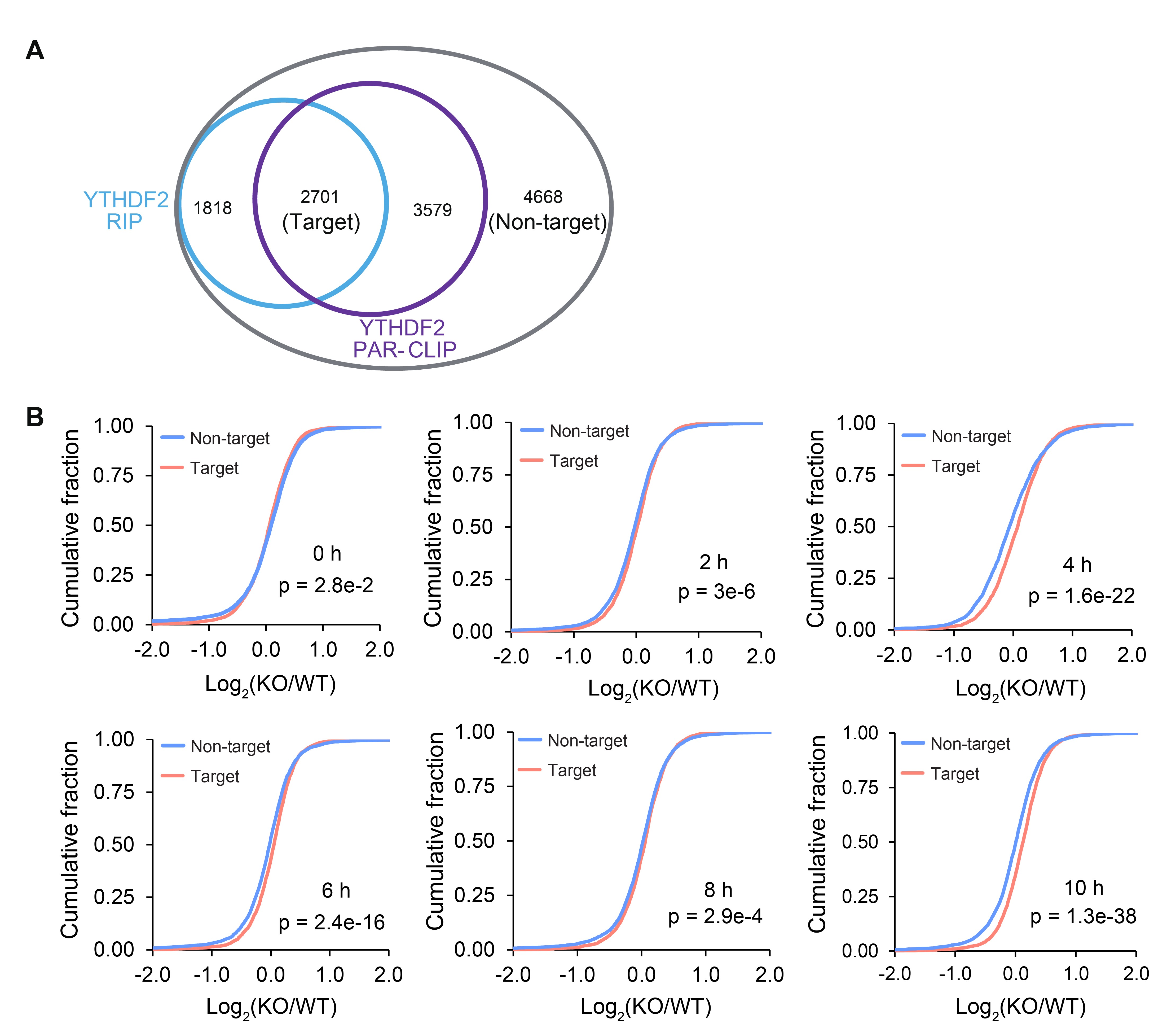

Supplement: S4 Fig — (A) Intersection for a confident YTHDF2 targets in HeLa cells between YTHDF2 RIP-seq and PAR-CLIP data. PAR-CLIP results are from Wang and colleagues [10]. The 4,668 nontarget genes were obtained after filtering out the genes in either RIP-seq or PAR-CLIP list and the ones with FPKM < 1 in the input sample of the RIP-seq data. (B) Cumulative distribution of 2,701 YTHDF2 targets and 4,668 nontargets by comparing WT and knockout cell lines. Genes with FPKM < 1 at each time point were further removed from the analysis. x-Axes indicate the log2 fold change of gene expression in knockout versus wild type. P values were calculated using the Mann-Whitney test. Underlying data for this figure can be found in S1 Data. FPKM, Fragments Per Kilobase of transcript per Million mapped reads. (TIF) [file pbio.3000664.s004.tif]

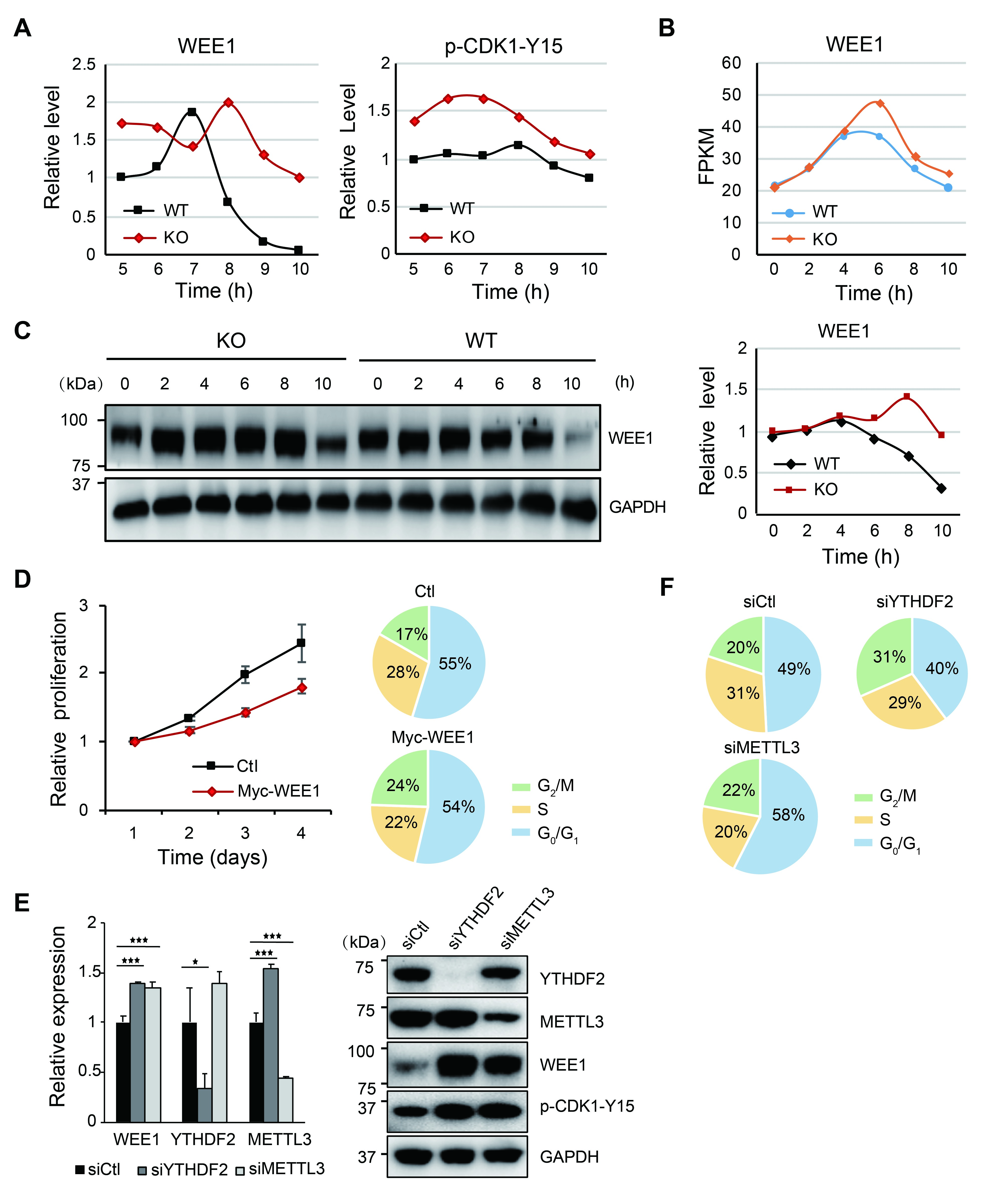

Supplement: S5 Fig — (A) Quantification of WEE1 and p-CDK1-Y15 by ImageJ from Fig 3B. The protein levels were normalized to the loading control GAPDH. (B) Expression level of WEE1 revealed by RNA-seq in wild-type and knockout cells at different time points post release from G1/S phase. (C) Western blot of WEE1 at different time points post synchronization in wild-type and YTHDF2 knockout HeLa cells. The right panel shows the normalized values of WEE1 quantified by ImageJ. (D) Effect of WEE1 overexpression in HeLa cells. Left panel shows cell proliferation of HeLa cells transfected with Myc-WEE1 compared with the empty vector control. The right panel shows flow cytometry analysis results of each phase during cell cycle. The percentages of each phase were quantified using FlowJo. (E) siRNA knockdown of YTHDF2 and METTL3 in HeLa cells. The left panel shows RT-qPCR results with two-sided Student t test (*P < 0.05; **P < 0.01; ***P < 0.001). The right panel shows western blot results of each protein. (F) Flow cytometry results of each phase in the cell cycle upon YTHDF2 or METTL3 knockdown. Underlying data for this figure can be found in S1 Data and S1 Raw Images. (TIF) [file pbio.3000664.s005.tif]

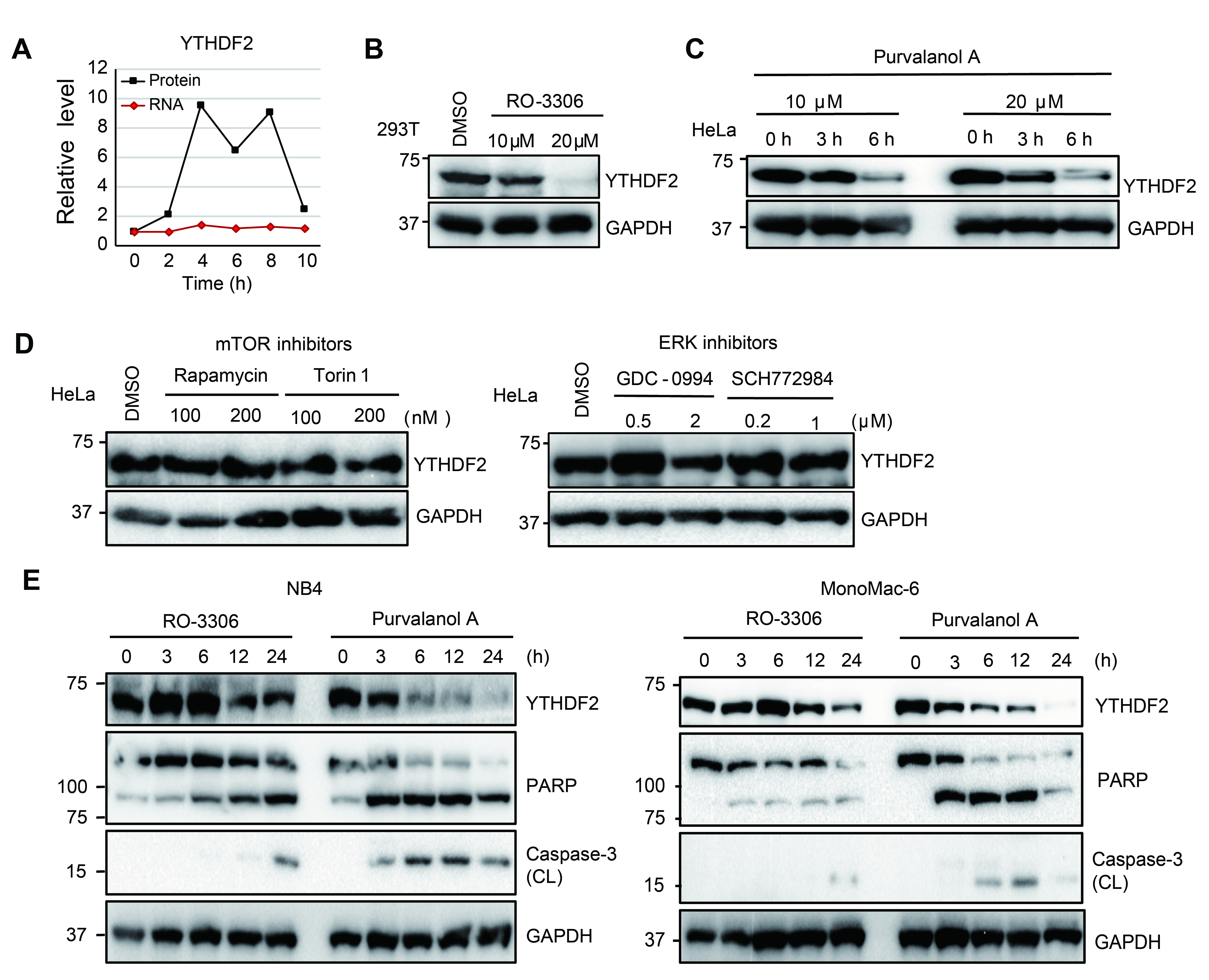

Supplement: S6 Fig — (A) The level of YTHDF2 at different time points post synchronization. The black line indicates protein level changes of YTHDF2 quantified by ImageJ. The red dots indicate transcript levels of YTHDF2 at each time point, which were normalized to the value at 0 hours. (B) RO-3306 induces YTHDF2 degradation in HEK 393T cells within 3 hours. The concentrations of RO-3306 are indicated. (C) Purvalanol A, a different CDK1 inhibitor, induces YTHDF2 degradation. Concentrations and incubation times are as indicated. (D) Detection of YTHDF2 levels after the treatment of relevant inhibitors, including mTOR and ERK inhibitors. (E) Western blot of YTHDF2 and apoptosis markers PARP and Caspase-3 (cleaved form) after CDK1 inhibitor treatment in NB4 and MonoMac-6 cells. Underlying data for this figure can be found in S1 Data and S1 Raw Images. (TIF) [file pbio.3000664.s006.tif]

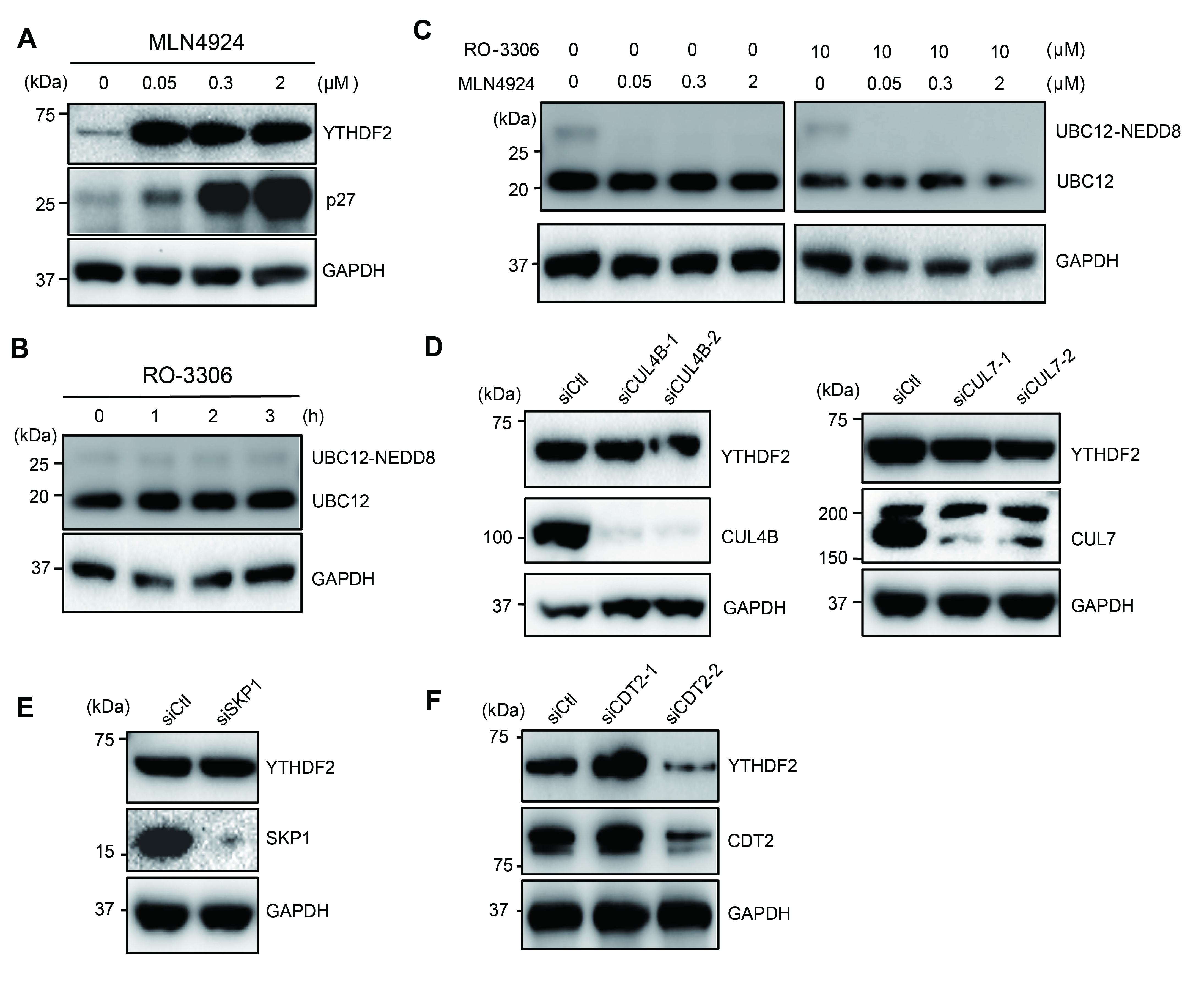

Supplement: S7 Fig — (A) Western blot of YTHDF2 after cells incubated with different concentrations of MLN4924 for 24 hours. p27 is a positive control. (B) Detection of the conjugated form of UBC12-NEDD8 after sequential treatment of RO-3306 and MLN4924. Cells were pretreated with RO-3306 for 3 hours before MLN4924 incubation for 3 hours, with concentrations indicated in the figure. (C) Detection of the conjugated form of UBC12-NEDD8 after treating cells with 10 μM RO-3306 for indicated hours. (D–F) Western blot of YTHDF2 after siRNA knockdown of CUL4B, CUL7, SKP1, and CDT2. Underlying data for this figure can be found in S1 Raw Images. (TIF) [file pbio.3000664.s007.tif]
