# Supplementary material for: YTHDF2 promotes mitotic entry and is regulated by cell cycle mediators
Source: PLoS Biol. 2020 Apr 8;18(4):e3000664. doi: 10.1371/journal.pbio.3000664 (PMC7170294; doi:10.1371/journal.pbio.3000664)

Raw images of western blotting

Related to Fig 2A

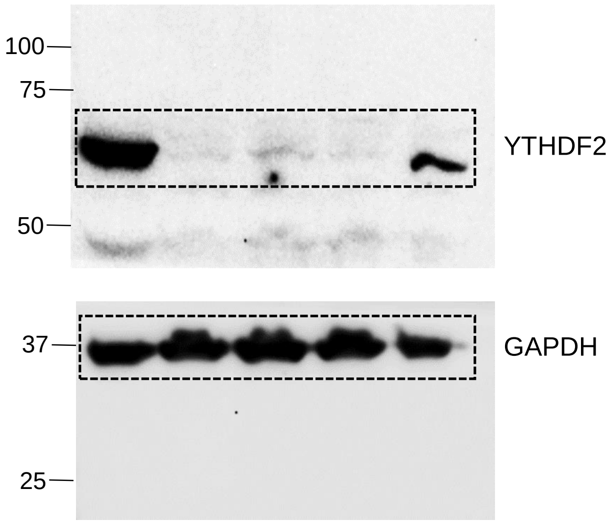

Related to Fig 3B

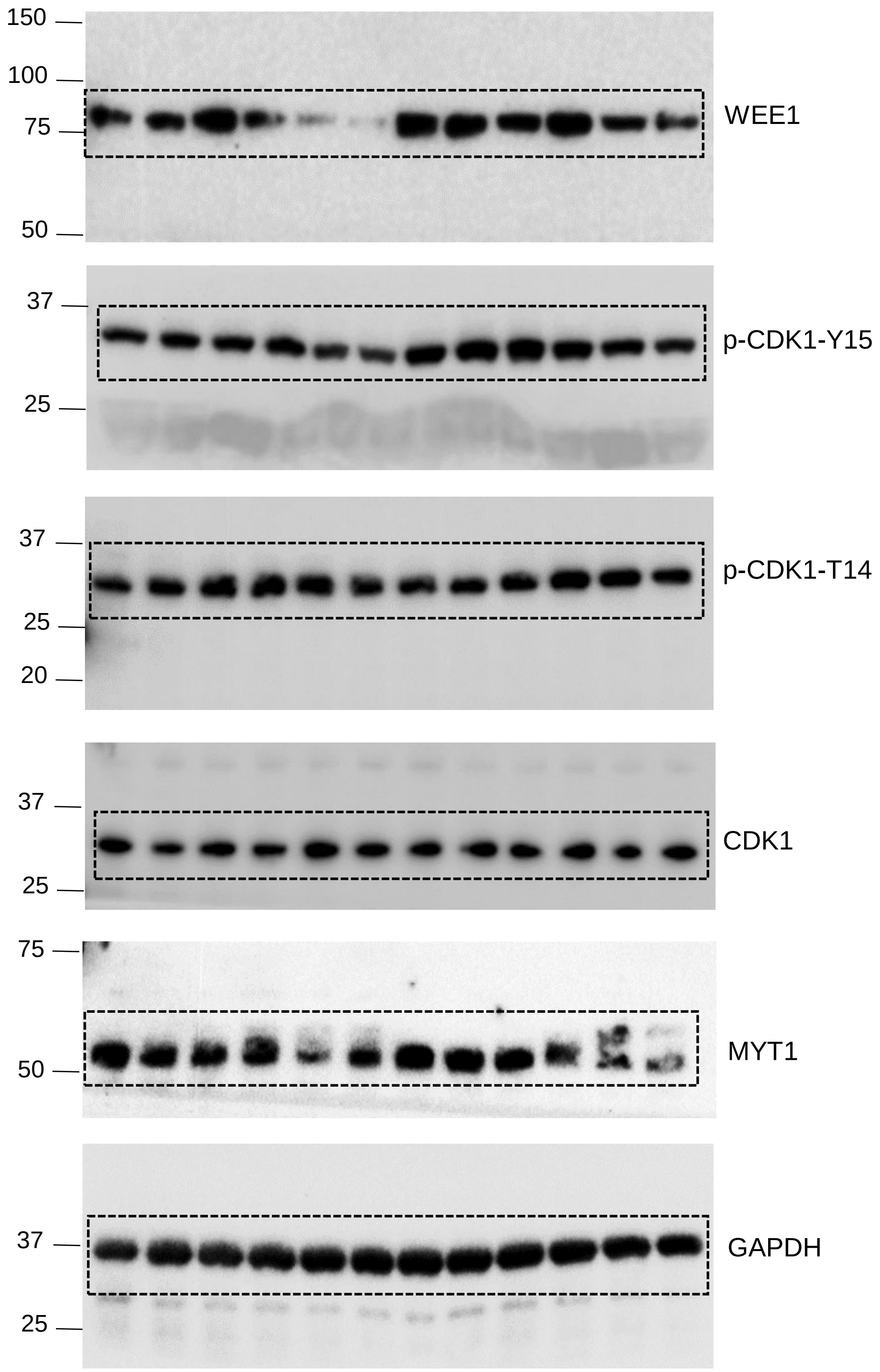

Related to Fig 4A

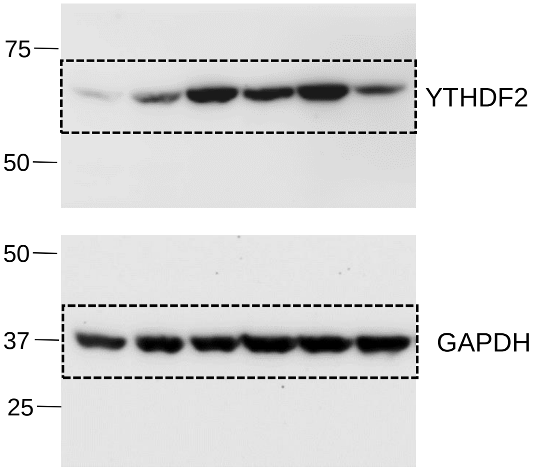

Related to Fig 4B

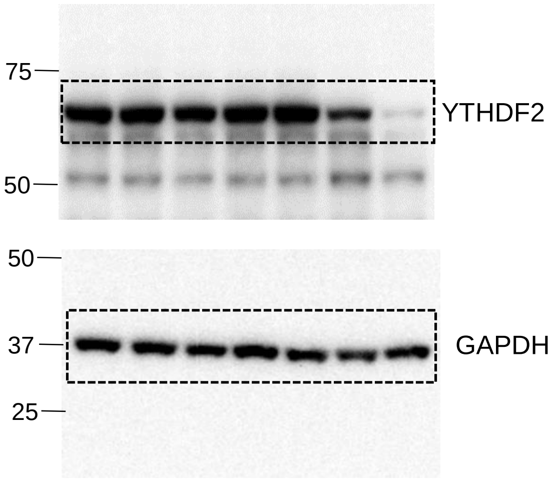

Related to Fig 4C

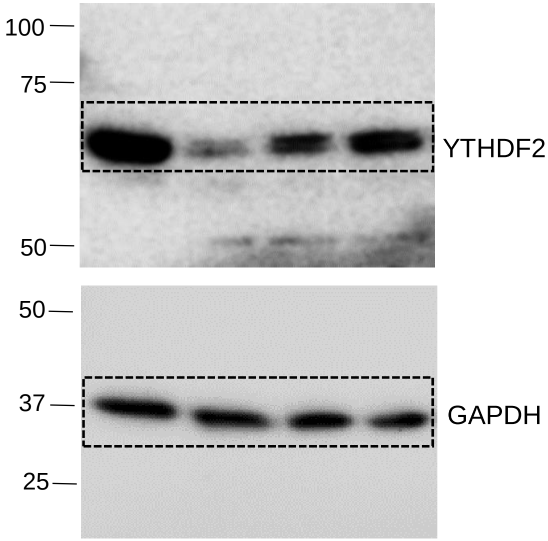

Related to Fig 4D

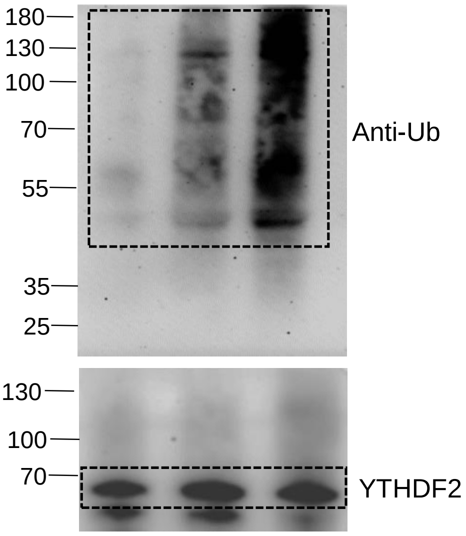

Related to Fig 4E

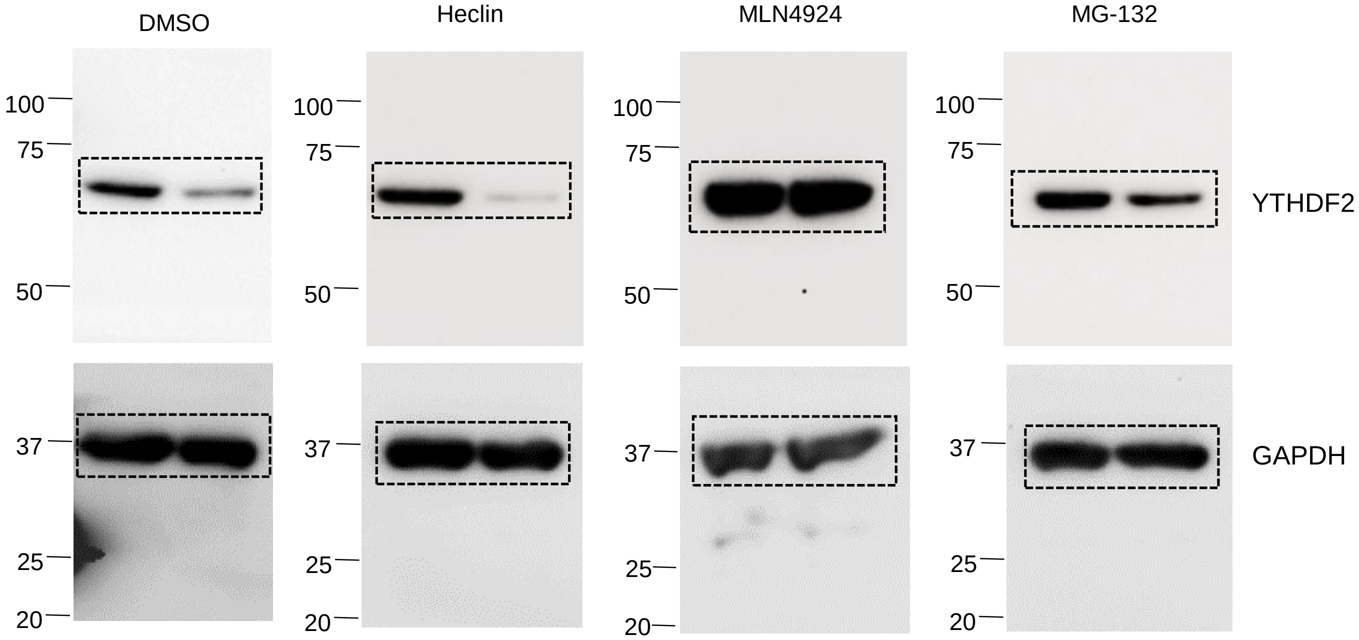

Related to Fig 4F

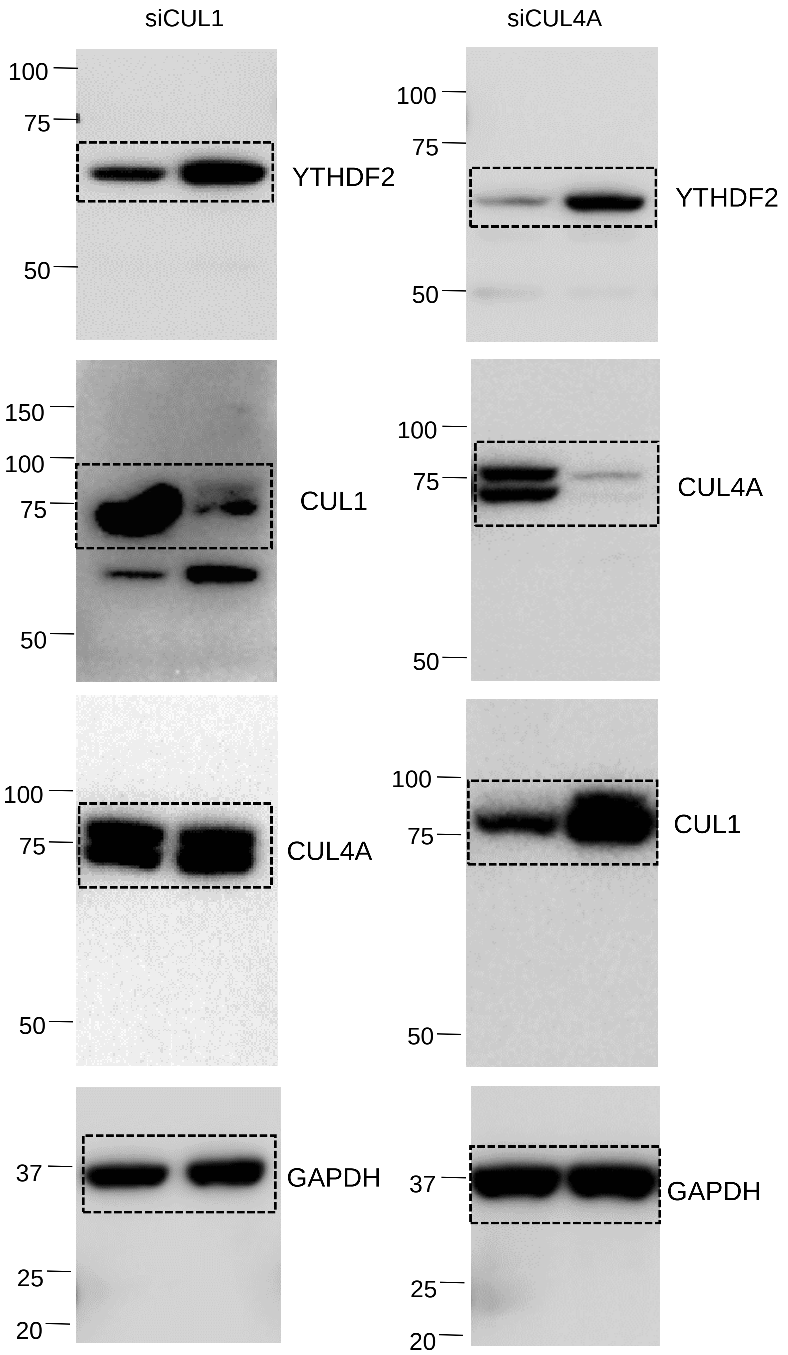

Related to Fig 4G

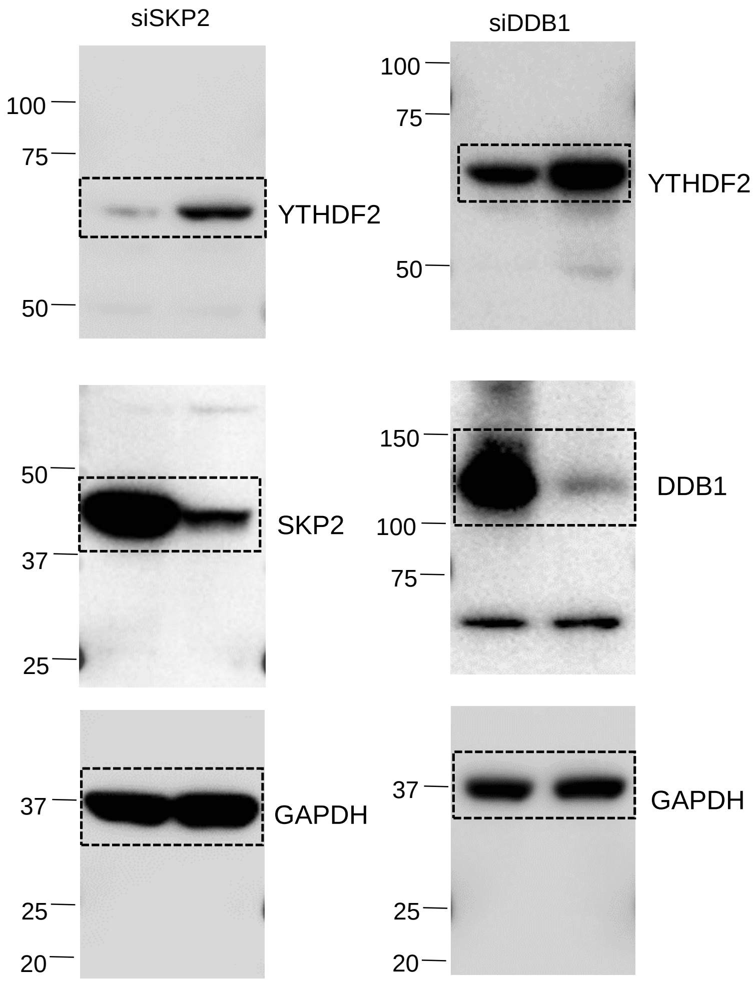

Related to Fig 4H

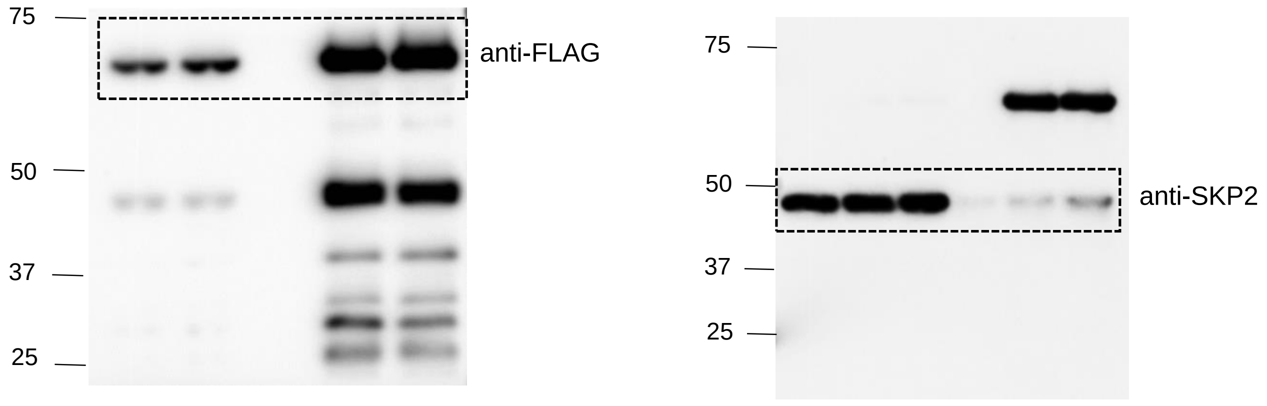

Related to Fig S4E

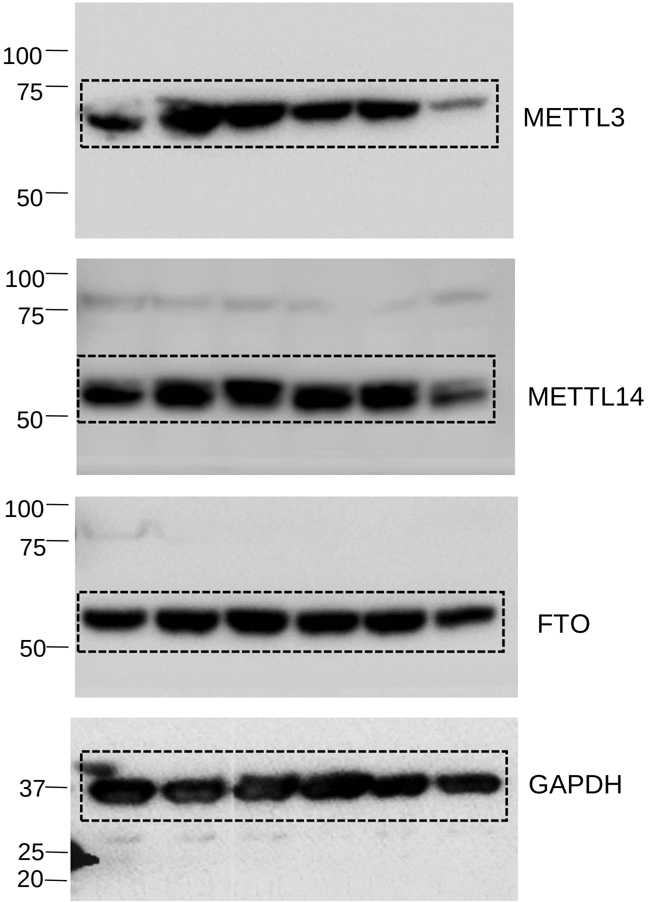

Related to Fig S5C

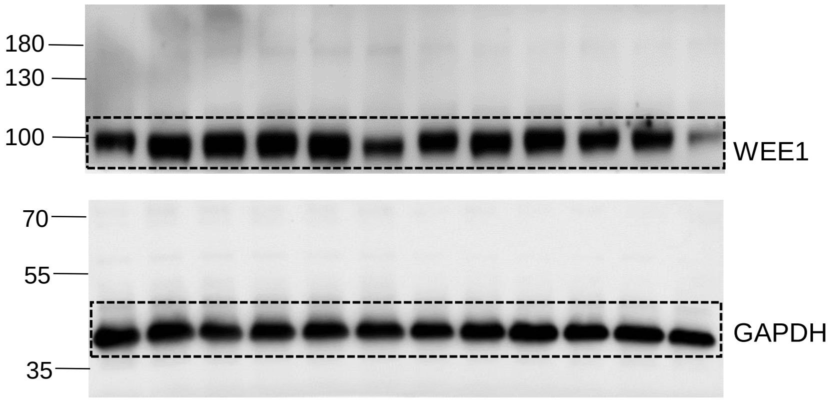

Related to Fig S5E

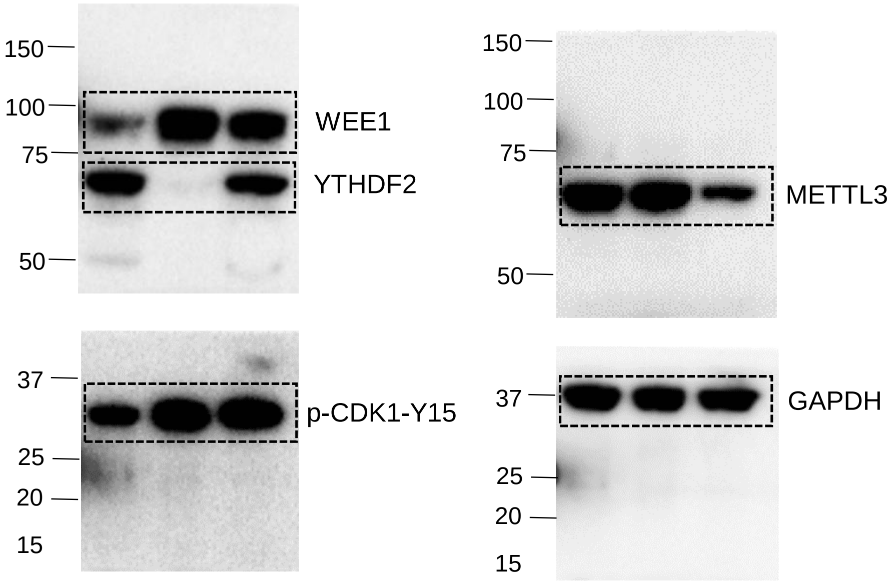

Related to Fig S6B

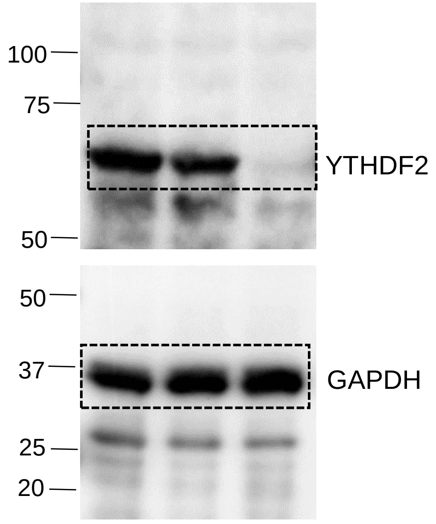

Related to Fig S6C

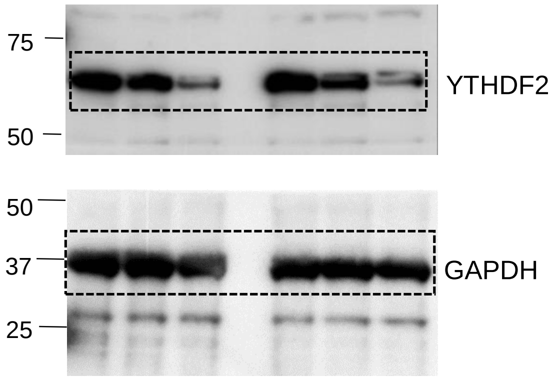

Related to Fig S6D

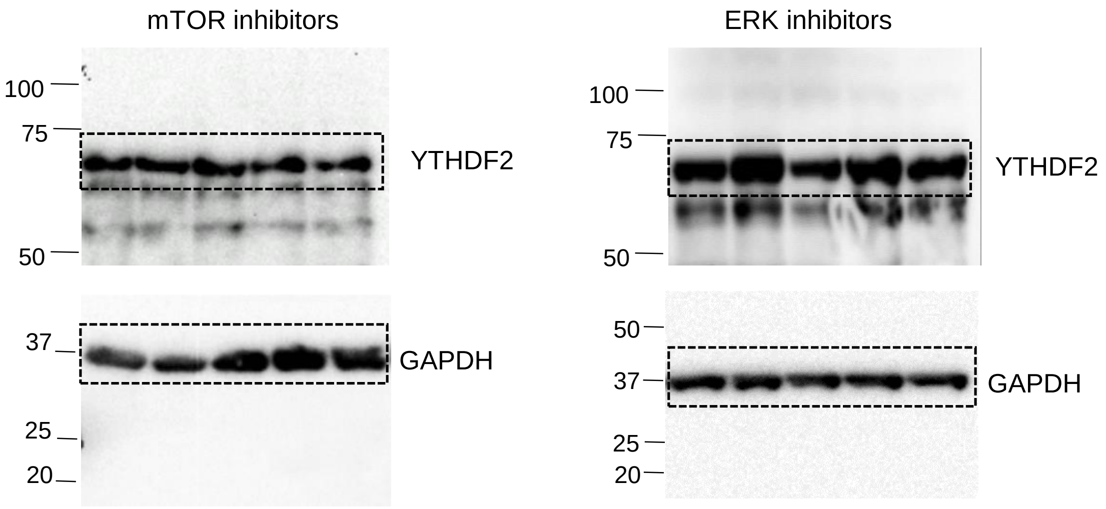

Related to Fig S6E

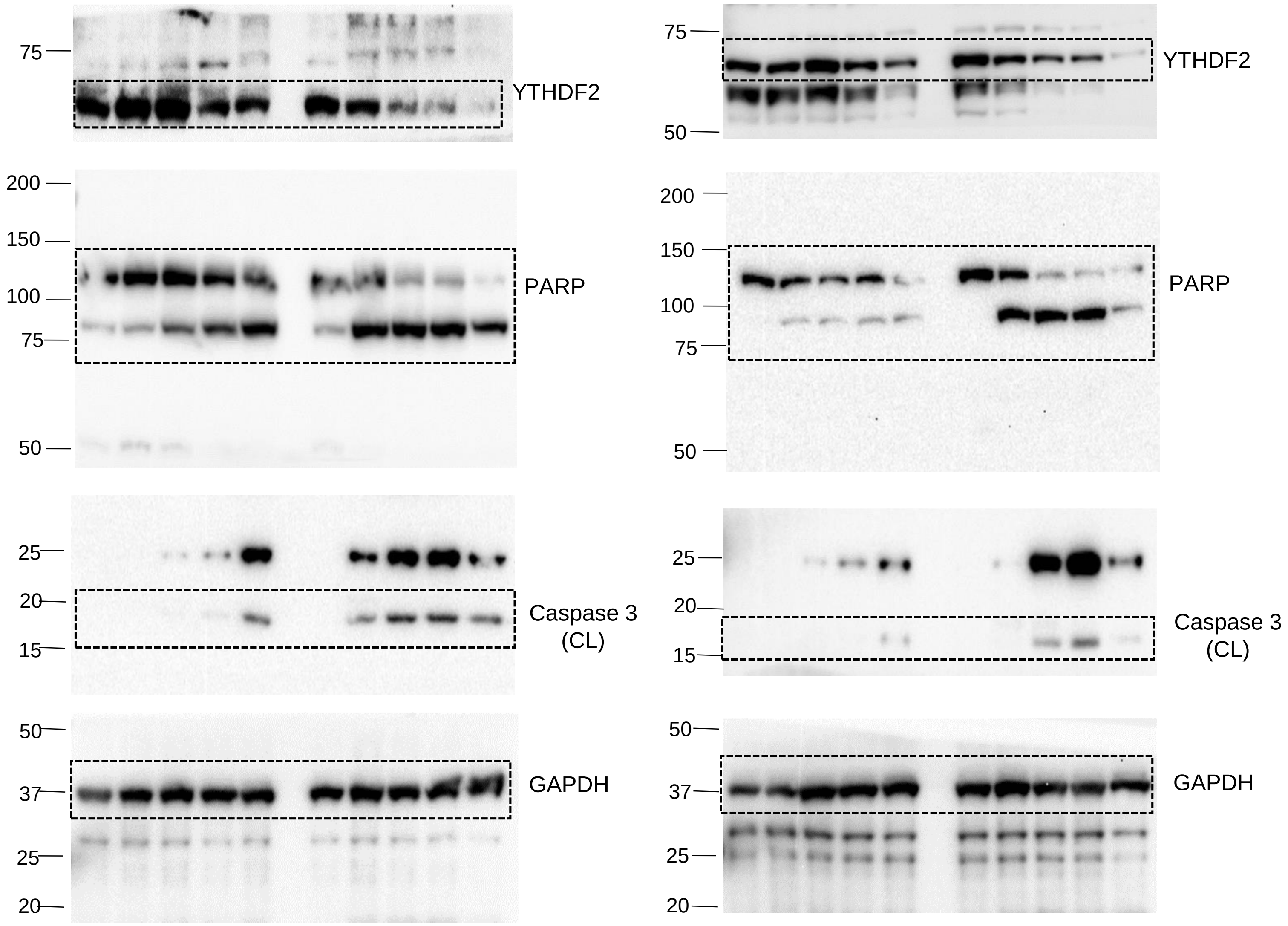

Related to Fig S7A

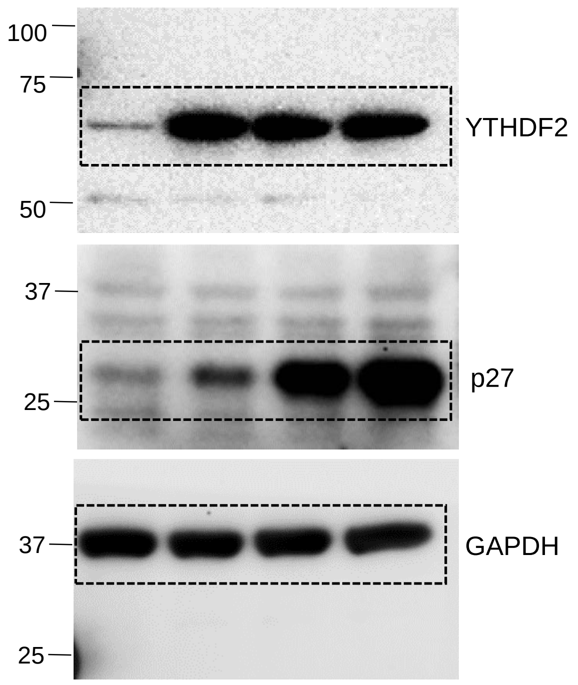

Related to Fig S7B

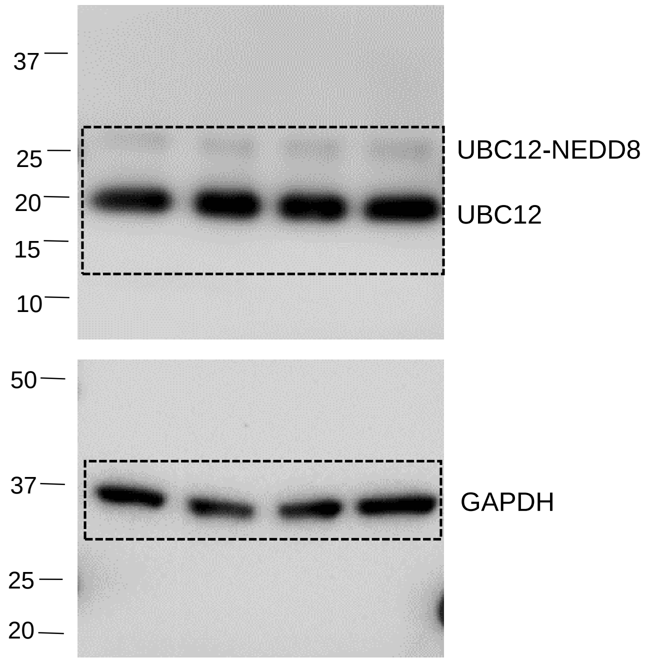

Related to Fig S7C

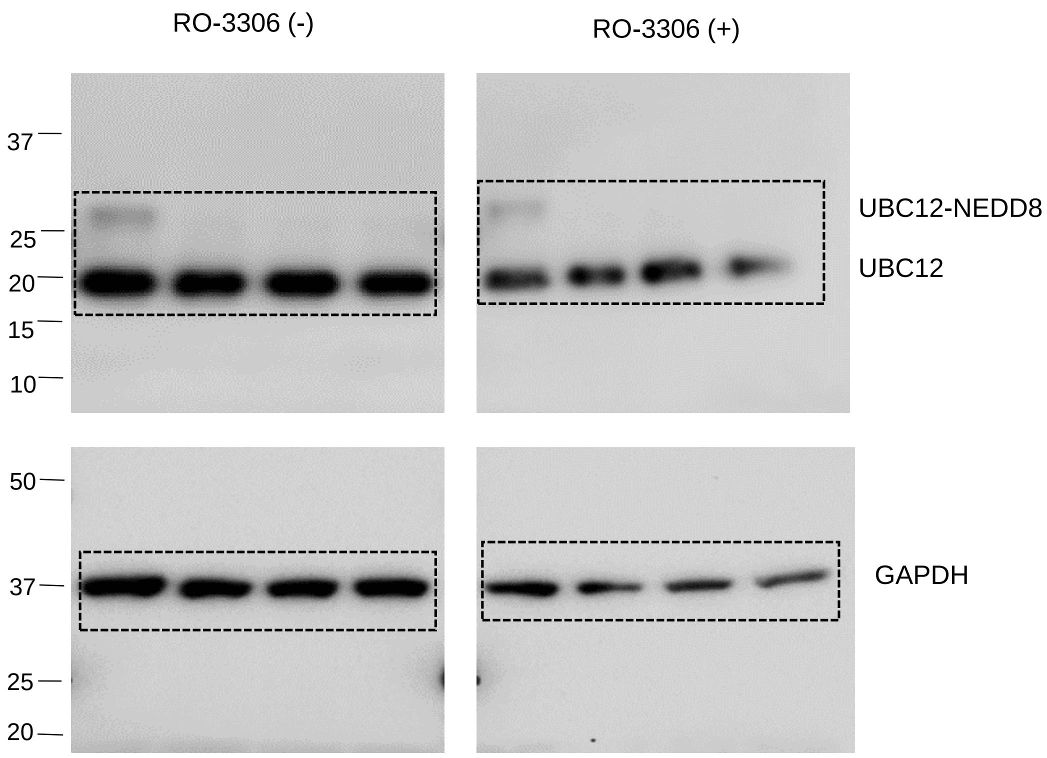

Related to Fig S7D

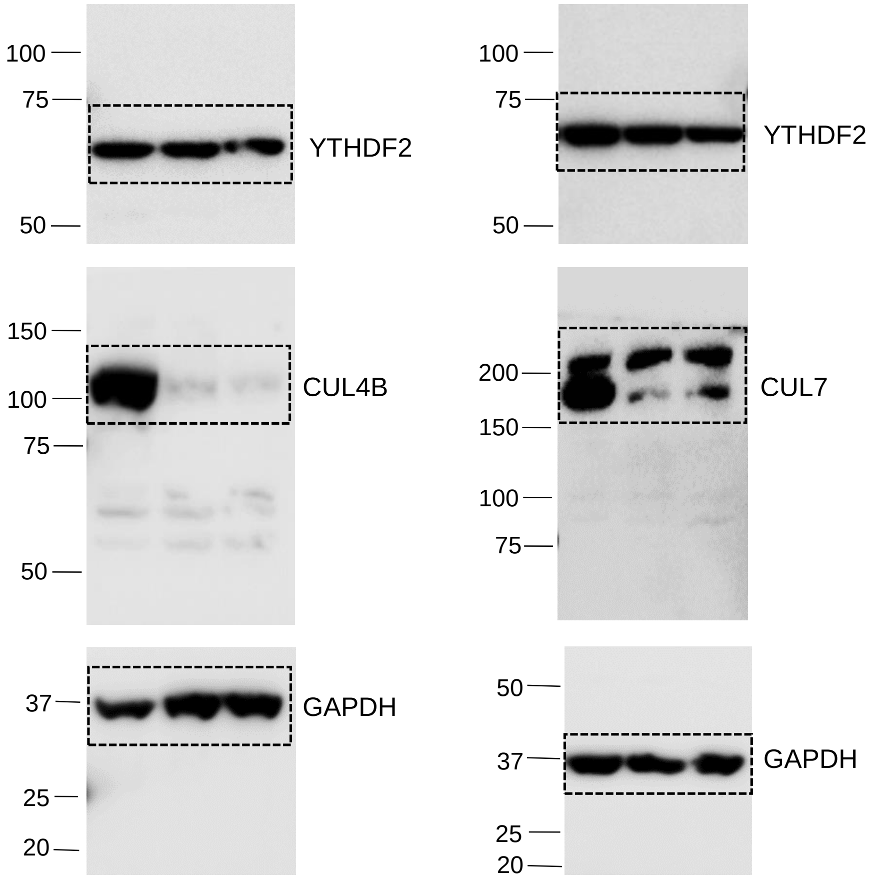

Related to Fig S7E

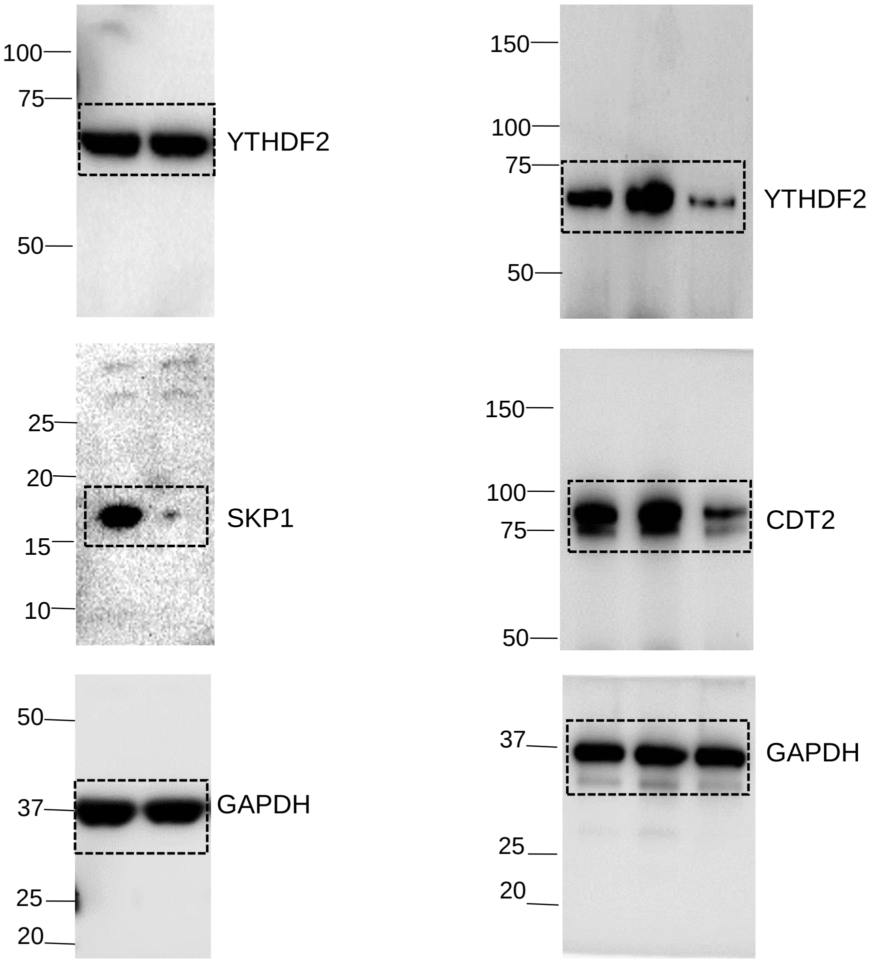

Supplement: S1 Raw Images — (PDF) [file pbio.3000664.s014.pdf]
